# Unprecedented Ultra‐High Expansion Ratio Foam for Innovative Architecture

**DOI:** 10.1002/advs.202501188

**Published:** 2025-03-24

**Authors:** Wenyu Zhong, Yichong Chen, Dongdong Hu, Jiayang Sun, Xingyu Jia, Ling Zhao

**Affiliations:** ^1^ State Key Laboratory of Chemical Engineering Shanghai Key Laboratory of Multiphase Materials Chemical Engineering School of Chemical Engineering East China University of Science and Technology Shanghai 200237 P. R. China; ^2^ Shanghai Electronic Chemicals Innovation Institute East China University of science and Technology Shanghai 201419 P. R. China

**Keywords:** conduction‐microwave heating assisted CO_2_ foaming, negative Poisson's ratio, passive daytime radiant cooling (PDRC) materials, PMMA/PVDF, ultra‐high expansion ratio foam

## Abstract

Global climate warming has dramatically increased the demand for space cooling. Materials that integrate superior thermal insulation with passive daytime radiative cooling properties hold significant promise for reducing energy consumption for space cooling during hot summers. In this study, conduction‐microwave heating assisted CO_2_ foaming process is used to optimize cell size and expansion ratios, producing PMMA/PVDF foam with an ultra‐high expansion ratio of 120 times and small, uniform cells. The foam is hydrophobic, chemically resistant, and recyclable, with a negative Poisson's ratio structure that gives it outstanding compression strength, elasticity, and flexibility, making it suitable for both everyday use and extreme weather conditions. The inherent properties of the material and its cell structure confer low thermal conductivity (26.69 mW m^−1^ K^−1^), high solar reflectance (96.37%), and high infrared emissivity (97.34%). This means that indoor cooling of buildings can be achieved in hot weather (15 °C difference in test results before and after use), meeting the cooling needs of buildings in most countries around the world. The ultra‐high expansion ratio PMMA/PVDF foam demonstrates significant potential in energy conservation, reducing carbon footprints, and promoting sustainability, providing a solution for the development of next‐generation buildings.

## Graphical abstract

PMMA/PVDF foams with ultra‐high expansion ratios are achieved through a microwave‐enhanced foaming process. The unique combination of matrix material properties and cell structure confers these foams with exceptional radiative cooling efficiency and thermal insulation performance, making them promising candidates for next‐generation passive radiative cooling systems in green building applications.

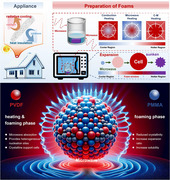

## 1 Introduction

Global climate warming has dramatically increased the demand for space cooling. Over the past 30 years, carbon dioxide (CO_2_) emissions from space cooling have reached 1 billion metric tons.^[^ ^1^ ^]^ Currently, active cooling systems for building thermal comfort, such as air conditioners, account for over 10% of global greenhouse gas emissions.^[^ ^2^ ^]^ The carbon footprint of space cooling is further exacerbating global warming, creating a vicious cycle. Passive daytime radiative cooling (PDRC) strategies offer a perfect alternative to traditional space cooling technologies. PDRC can cool buildings by dissipating heat to the cold outer space through the atmospheric long‐wave infrared transparency window (8–13 µm) without consuming energy or emitting greenhouse gases. Raman et al.^[^ ^3^ ^]^ developed a radiative cooler consisting of seven layers of HfO_2_ and SiO_2_ that reflects 97% of incident sunlight while emitting selectively and strongly within an atmospheric transparent window. However, conventional radiative cooling materials often neglect thermal insulation performance in their structural design. In hot summer months, the significant indoor‐outdoor temperature difference can greatly increase non‐radiative heat flux transfer through convection and conduction, thereby severely affecting cooling performance and significantly raising energy consumption.^[^ ^4^ ^]^ Integrating thermal insulation properties into radiative coolers can simultaneously prevent outdoor heat from entering indoors and provide additional cooling. This approach offers a forward‐looking solution for designing efficient building cooling and insulation structures.

High‐standard design of building requires radiative cooling materials to possess high solar reflectance (${\bar{R}}_{solar}$), high infrared emittance (${\bar{\varepsilon }}_{LWIR}$), and low thermal conductivity. These properties ensure the material can reflect solar energy back into outer space while minimizing active heat exchange between the outdoor environment and the building. Passive radiative cooling strategies are very common in nature, evident in plant leaves,^[^ ^5^ ^]^ animal fur,^[^ ^6^ ^]^ and insect shells.^[^ ^1^ ^]^ Harnessing the power of nature provides valuable inspiration for advanced PDRC designs. Inspired by the microstructures on the wings of the Central American butterfly “Morpho theseus”^[^ ^7^ ^]^ and the stem surface of the China bamboo fungus “Phallus indusiatus”,^[^ ^8^ ^]^ which effectively reflect sunlight, designing radiative cooling materials with porous structures can enhance solar light scattering within the solar radiation spectrum, making it a viable solution for building applications. Polymers contain abundant chemical bonds capable of absorbing infrared radiation,^[^ ^9^ ^]^ and their inherently high infrared emittance makes them an ideal raw material for radiative coolers. Therefore, constructing porous structures on the surface of polymers can further enhance their solar reflectance, while creating a porous structure inside the polymer can effectively inhibit heat flow transfer. The coupling of above two approaches enables the material to fully meet the requirements of low‐carbon, energy‐efficient, and sustainable building applications.

CO_2_ foaming represents an innovative, eco‐friendly, and sustainable porous‐making technology poised to enable the preparation of foams with high expansion ratio,^[^ ^10^ ^]^ meeting the synergistic requirements for both radiative cooling properties and high thermal insulation properties of radiant coolers Building upon this technology, current innovative processes for producing foams with high expansion ratio commonly involve modifying the polymer matrix,^[^ ^11^ ^]^ introducing co‐blowing agents,^[^ ^12^ ^]^ and employing external‐field enhancement techniques.^[^ ^13^, ^14^ ^]^ For instance, Yang et al.^[^ ^13^ ^]^ successfully achieved polystyrene (PS)/poly(methyl methacrylate) (PMMA)/carbon nanotubes (CNTs) composite foams with an ultrahigh expansion ratio exceeding 80 times through the synergistic effect of ultrasonic field enhancement and co‐blowing agents. These processes aim to enhance the nucleation, growth, and shaping of cells within the polymer matrix by altering polymer properties, as well as the solubility and diffusion properties of the blowing agent. However, the limited increase in expansion ratio is not sufficient to meet the demand for ultra‐lightweight and high‐end applications. Additionally, there have been no reports of foams exceeding an expansion ratio of 100 times utilizing CO_2_ foaming technology. As a novel heating method, microwave can induce high‐frequency vibrations in polar molecules.^[^ ^15^ ^]^ The resulting losses from these vibrations efficiently and rapidly heat the material. Therefore, the application of microwave‐enhanced technology in the CO_2_ foaming process, compared to traditional temperature‐raising foaming, can achieve more uniform temperature distribution and longer foaming time, potentially resulting in high‐expansion foams. Based on this, our team has innovatively proposed a conductive‐microwave synergistic heating‐assisted foaming process to efficiently prepare PS and thermoplastic polyurethane elastomer (TPU) foams with high expansion ratios by means of dual heating inside and outside of the polymer.^[^ ^16^, ^17^ ^]^ This method offers a promising solution for endowing radiant coolers that possess high infrared emissivity with high expansion ratios, excellent thermal insulation, and superior solar reflection properties.

**Figure 1:**
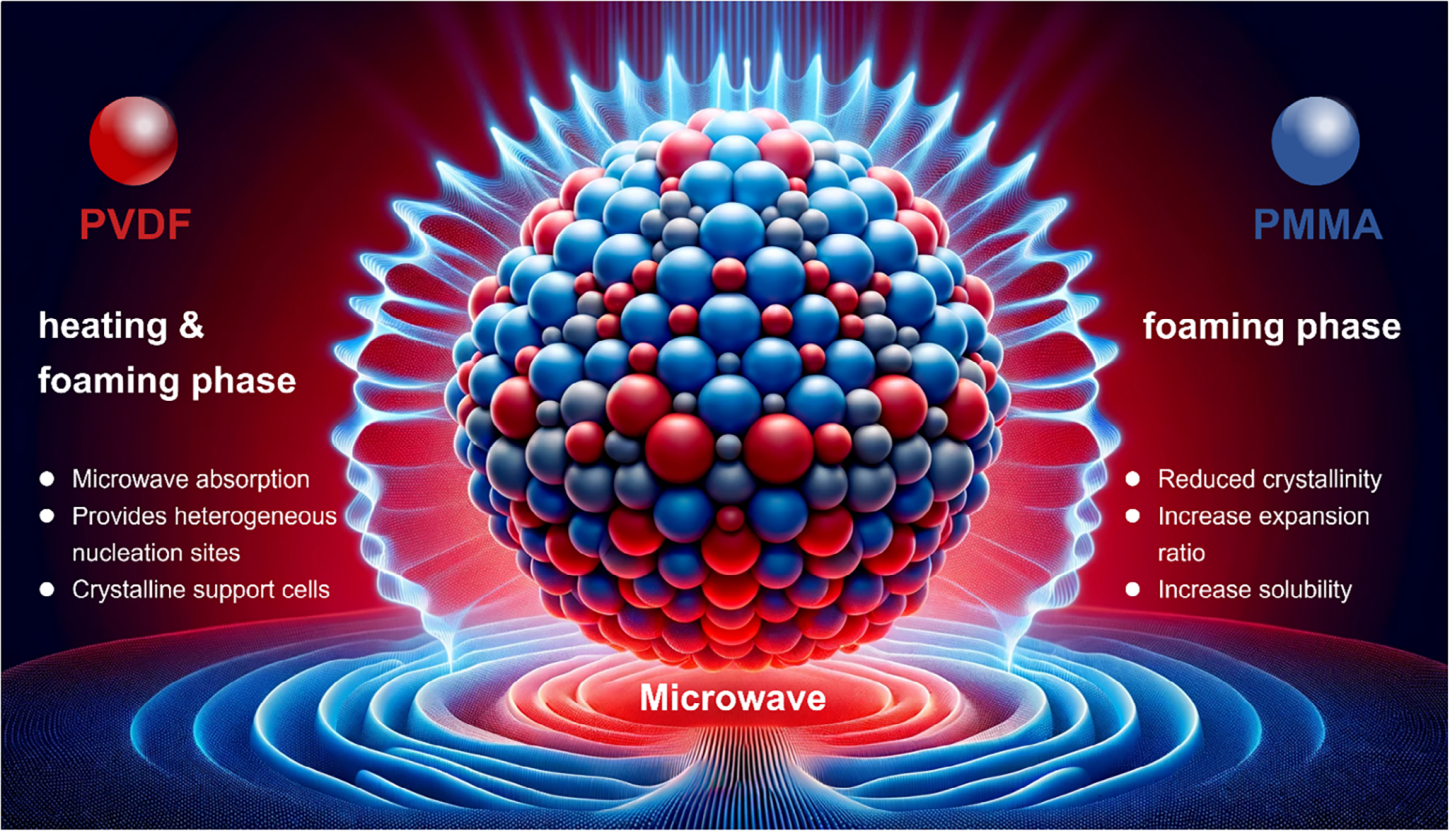
Schematic Diagram of The Complementary Mechanism of PMMA and PVDF.

PMMA is a typical “microwave transparent” material,^[^ ^18^ ^]^ making it unsuitable for microwave‐assisted heating foaming processes. A feasible approach to overcome this limitation is to incorporate a microwave‐absorbing polymer into the polymer matrix, thereby enabling the heating of the whole system under microwave irradiation. Poly(vinylidene fluoride) (PVDF), with its highly polar groups^[^ ^19^ ^]^ and excellent compatibility with PMMA,^[^ ^20^ ^]^ is a promising candidate for facilitating the microwave foaming of PMMA (**Figure** **1**). Despite numerous attempts in depressurization foaming and traditional temperature rising foaming,^[^ ^20^ ^]^ the unsatisfactory expansion ratio has limited the application of this system. By adjusting material ratios, saturation conditions, and foaming parameters, the crystallinity of the matrix, the size of the crystals, and the solubility of CO_2_ in the polymers can be synergistically adjusted. This approach led to the preparation of ultra‐high expansion ratio PMMA/PVDF foams featuring small, uniform cells. An initial expansion ratio exceeding 200 times and a stabilized expansion ratio of 120 times were achieved. The resultant foams with high expansion ratio exhibit hydrophobicity and chemical resistance. Moreover, the negative Poisson's ratio structure formed by the rapid shrinkage of the foam endows it with superior compression performance, high elasticity, and excellent flexibility. These properties make the foams suitable for daily use and capable of withstanding harsh outdoor conditions, demonstrating exceptional durability. The microcellular structure in the PMMA/PVDF foams, along with the inherent molecular characteristics of the materials, endows it with low thermal conductivity, high solar reflectance, and high infrared emissivity. Compared to traditional thermal insulation building materials, these foams offer superior thermal insulation and PDRC performance. This makes them highly promising for next‐generation outdoor building materials and suggests their significant potential in energy‐efficient cooling and low‐carbon emission applications.

## 2 Results and Discussion

### 2.1 Basic Physical Properties of PMMA/PVDF

**Figure 2:**
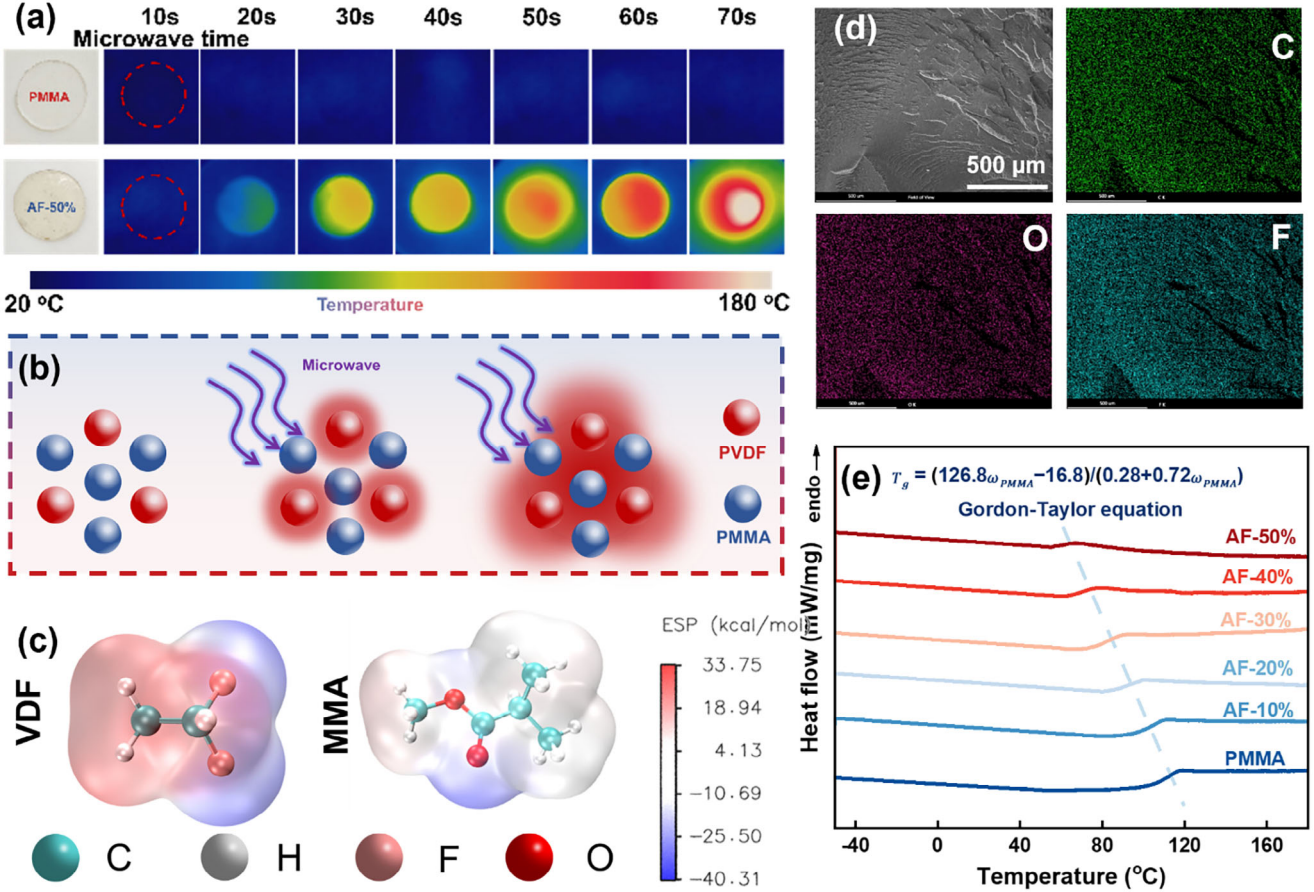
Basic Properties of PMMA/PVDF. a) Infrared thermal imaging of PMMA and AF‐50% heated under 1000 W microwave radiation at different times; b) The microwave heating mechanism of the PMMA/PVDF blend system; c) Electrostatic representations of VDF and MMA; d) Elemental distribution energy spectrum of AF‐50%; e) DSC melting curves for PMMA and PMMA/PVDF blends.

The high polarity of PVDF endows it with efficient microwave heating characteristics,^[^ ^19^ ^]^ making microwave‐assisted foaming of PMMA/PVDF blends feasible. **Figure** **2**a depicts the temperature variation of PMMA and AF‐50% after radiating at microwave power of 1000 W for different times. Pure PMMA, which is almost non‐absorptive to microwaves, shows no significant changes even after prolonged microwave irradiation. In contrast, PMMA/PVDF blended system exhibits a significant and sustained temperature increase. The reason lies in the PVDF molecular chains within the PMMA/PVDF blend, which can rapidly absorb microwave energy and convert it into heat. This heat is then transferred to the PMMA molecular chains through thermal conduction, enabling the overall macroscopic microwave heating of the blend system (Figure 2b).

The polarity of polymers is a key indicator for evaluating their microwave heating characteristics. Generally, highly polar materials exhibit uneven charge distribution and heat up more quickly under microwave radiation, while weakly polar materials show the opposite behavior. The electrostatic potential (ESP) of a material can reflect the distribution of charges within its molecules, making it an effective tool for assessing the polarity of PMMA and PVDF and their interactions with microwaves. The ESP isosurfaces of vinylidene fluoride (VDF) and methyl methacrylate (MMA) were calculated using density functional theory (DFT). As shown in Figure 2c, the fluorine (F) and hydrogen (H) atoms in VDF molecules exhibit highly asymmetric charge distribution. The strong dipoles formed by the C‐F and C‐H bonds result in significant positive and negative ESP values, indicating that PVDF has strong polarity and a high dielectric constant, which facilitates efficient microwave heating. In contrast, MMA shows only weak negative ESP at the ester group (‐COO‐), resulting in a relatively uniform overall charge distribution. This demonstrates that PMMA has weak polarity and, under the same microwave irradiation conditions, exhibits lower microwave absorption capability. The ESP calculations provide a microscopic explanation for the heating mechanism in the PMMA/PVDF blend system, further confirming that the introduction of PVDF enables effective microwave‐assisted foaming of the blend.

For the PMMA/PVDF blend system, compatibility is crucial to determine heating uniformity and foaming behavior. Figure 2d shows the cross‐sectional morphology and elemental analysis of the AF‐50% sample. The microstructure of the PMMA/PVDF blend does not exhibit phase‐separated structures such as sea‐island or continuous‐phase regions. The elemental analysis reveals that the characteristic elements of PMMA (oxygen, O) and PVDF (fluorine, F) are uniformly distributed throughout the system, indicating good compatibility between PMMA and PVDF. This compatibility is attributed to strong interactions between the methylene (CH_2_) groups in PVDF and the carbonyl (C = O) groups in PMMA, leading to tightly entangled molecular chains.^[^ ^20^ ^]^ As shown in Figure 2e, the DSC melting curves of PMMA and PMMA/PVDF blends demonstrate that the blends exhibit a single glass transition temperature (T_g_) within the test range, regardless of the mixing ratio. This further confirms that the melt‐blended composites form a homogeneous blend system, indicating good molecular‐level compatibility between PVDF and PMMA. Moreover, as the PVDF content increases, the T_g_ of the PMMA/PVDF blend decreases linearly, which shows the same trend as the Gordon‐Taylor equation.^[^ ^21^ ^]^ PMMA, because of its high T_g_, requires more energy during the solid‐phase temperature rising foaming. However, when the PVDF content reaches 50%, the T_g_ of the blend is ≈70 °C, which is ≈40 °C lower than that of pure PMMA. The plasticizing effect of PVDF means that the blend system has a lower processing temperature, which requires less energy to drive cell nucleation and growth during the foaming process.

### 2.2 Interaction Between PMMA/PVDF and CO_2_

**Figure 3:**
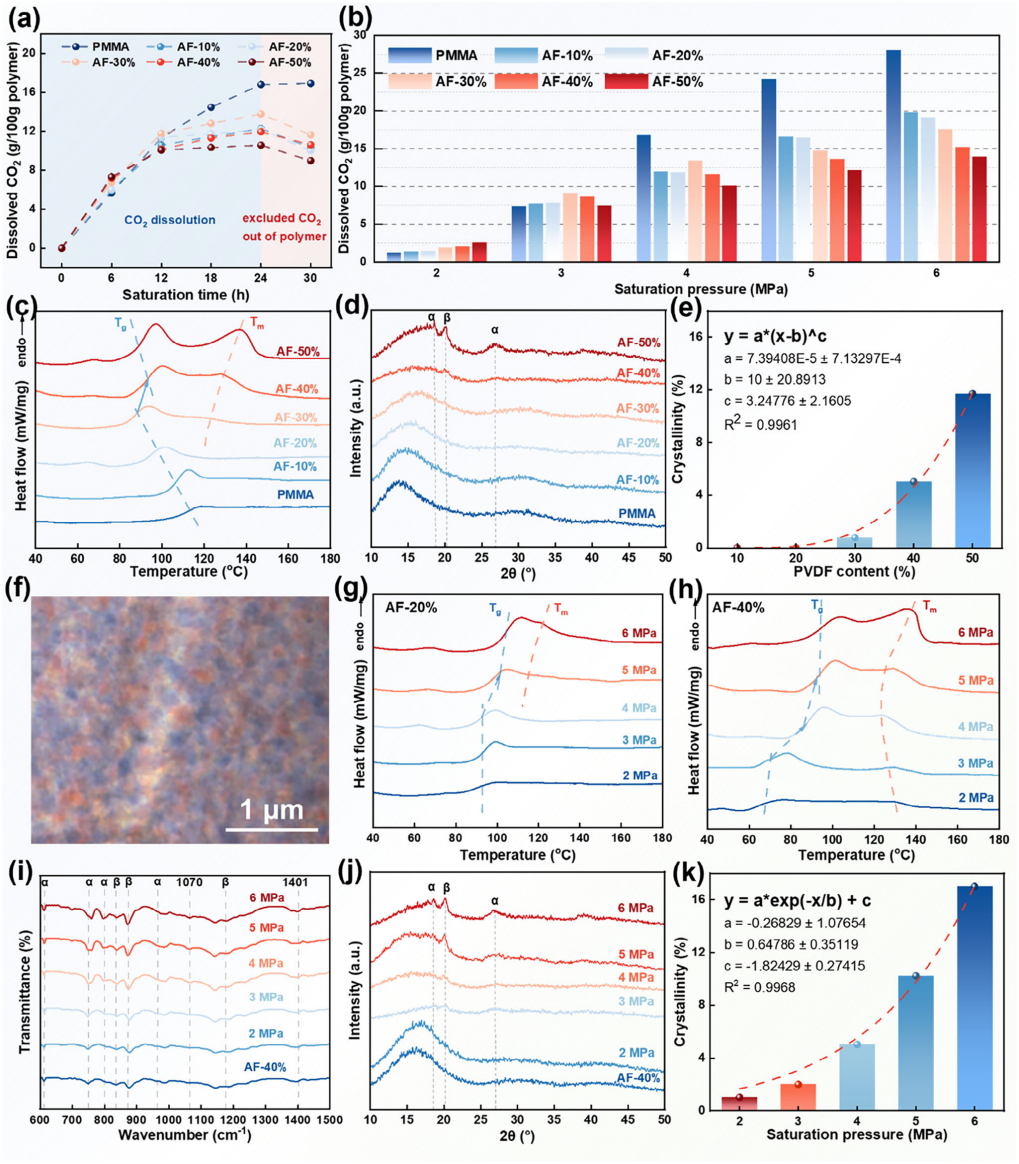
Interaction between PMMA/PVDF and CO_2_. a) CO_2_ solubility in PMMA and PMMA/PVDF after different saturation times at 4 MPa; b) CO_2_ solubility in PMMA and PMMA/PVDF after 24 h of saturation at different saturation pressures; c) DSC melting curves, d) XRD patterns, and e) crystallinity of PMMA and PMMA/PVDF after 24 h of saturation at 4 MPa CO_2_ (with gas fully diffused); f) POM image of AF‐40% after 24 h of saturation at 4 MPa CO_2_; g) DSC melting curves of AF‐20% after different saturation times (with gas fully diffused); h) DSC melting curves, i) FTIR spectra, j) XRD patterns, and k) crystallinity of AF‐40% after different saturation times (with gas fully diffused).

The solubility of CO_2_ in the PMMA/PVDF blends is crucial to evaluate the saturation conditions during solid‐state temperature rising foaming and to assess the foaming efficiency of the blends.^[^ ^22^ ^]^ **Figure** **3**a shows the variation in CO_2_ dissolved at different saturation times for blends with varying PVDF content at room temperature (25 °C) and under a CO_2_ pressure of 4 MPa. For the typical amorphous polymer PMMA, the amount of CO_2_ dissolved in the polymer increases with saturation time. This continues until the internal solubility and external gas pressure of the polymer reaches equilibrium upon complete saturation, at which point the CO_2_ solubility levels off.

For semi‐crystalline polymers PVDF, the plasticizing effect induced by the ingress of CO_2_ increases the free volume within the polymer matrix, thereby weakening intermolecular interactions and making the polymer chains more mobile.^[^ ^23^ ^]^ When the CO_2_ content within the polymer matrix reaches a certain threshold, the mobility of the polymer chains is substantially increased, leading to the crystallization of PVDF. It is generally accepted that the crystalline regions of polymers are difficult to dissolve in CO_2_. Consequently, during the crystallization process, the growth of crystals expels CO_2_ that was previously dissolved in the polymer matrix. At this stage, the solubility of CO_2_ in the blend containing PVDF is influenced by a competitive relationship between gas dissolution and crystallization‐induced expulsion. When the saturation time exceeds 24 hours, the rate at which crystallization expels CO_2_ surpasses the rate at which CO_2_ dissolves into the polymer matrix, resulting in a decreased CO_2_ solubility at prolonged saturation times (excluded CO_2_ out of polymer section in Figure 3a). Similar conclusions were drawn by Xia et al. in their study on PET.^[^ ^22^ ^]^ Furthermore, due to the differing affinities of PMMA and PVDF for CO_2_, the dissolution rate of CO_2_ and the rate at which crystallization expels CO_2_ vary significantly with different PVDF contents in the blends. This resulted in a remarkable regularity in dissolution of the blends at the same content ratio and disorder among the blends at different content ratios.

Since samples with different PVDF ratios exhibit the highest CO_2_ solubility after 24 h of saturation, subsequent heating and foaming processes are based on this saturation condition. Figure 3b illustrates the CO_2_ solubility of samples with varying PVDF contents after 24 h of adsorption under different saturation pressures. At low pressures, the plasticizing effect of PVDF on PMMA increases the free volume between molecular chains, thereby enhancing the solubility of the matrix to a certain extent. However, as the pressure increases, CO_2_ solubility in PMMA, which has a strong affinity for CO_2_ because of its ester groups, surpasses that in PVDF.^[^ ^24^ ^]^ Consequently, the solubility of polymers with high PVDF content tends to decrease. In addition, PVDF is more susceptible to crystallization under high pressure conditions.^[^ ^23^ ^]^ The crystallization induced by CO_2_ increases markedly with pressure, leading to the exclusion of CO_2_ from the polymer matrix and thus reducing solubility. This phenomenon, combined with the inherently lower solubility of PVDF compared to PMMA, results in a decrease in CO_2_ solubility as the PVDF content increases. Nevertheless, thanks to the CO_2_ affinity of PMMA, polymers with high PVDF content can still maintain relatively high solubility under high‐pressure conditions, which is beneficial for achieving effective foaming of the material.

The crystallization induced by CO_2_ has a serious impact not only the solubility of the polymer but also the nucleation and growth of cells during the foaming process. Therefore, it is essential to analyze the effect of CO_2_ atmosphere under different pressures on crystal formation. Figure 3c,d show the DSC melting curves and XRD patterns of PMMA and PMMA/PVDF after being saturated in a 4 MPa CO_2_ environment for 24 h. To prevent residual CO_2_ in the polymer from affecting the test results, the saturated samples were first stored at ‐40 °C (to prevent foaming) for one month and then at room temperature for another month to ensure complete diffusion of CO_2_ from the polymer matrix (ensuring the sample mass remained consistent before saturation and after diffusion). When the PVDF content is below 20%, neither the DSC nor the XRD curves show clear crystallization peaks, indicating that the low saturation pressure is insufficient to promote crystallization. However, when the PVDF content exceeds 30%, crystallization begins, as evidenced by the appearance of PVDF α‐phase diffraction peaks at 2θ = 17.8° and 26.2°, and β‐phase diffraction peaks at 2θ = 20.2° in the XRD patterns. Furthermore, as the PVDF content increases, the melting point (T_m_) of the blend starts to rise and the melting range widens, indicating that crystallization becomes more complete, crystal size increases, and crystal size distribution becomes more heterogeneous. Figure 3e displays the crystallinity of the polymer at different PVDF contents after saturation with 4 MPa CO_2_. PMMA can inhibit the crystallization of PVDF in the blend system. Therefore, the crystallinity of the polymer matrix only increases sharply when the PVDF content reaches a certain threshold. During this process, the crystallization ability of PVDF is significantly enhanced, leading to more orderly folding of PVDF molecular chains, resulting in more complete crystallization and larger crystal sizes.

The T_g_ of PMMA/PVDF blends is influenced by the amorphous regions of both PMMA and PVDF. Since the T_g_ of PVDF is significantly lower than that of PMMA, at low PVDF content, the T_g_ of the saturated polymer shows a trend similar to that of the unsaturated polymer. However, when the content of PVDF reaches 40%, its T_g_ shows a sudden increase, which is due to the formation of crystalline structures after CO_2_ saturation that creates a microphase separation within the polymer matrix (Figure 3f). As crystallization increases, the proportion of the amorphous regions in PVDF diminishes, thereby reducing the influence of PVDF on T_g_, making the T_g_ closer to that of PMMA. Figures 3g,h show consistent findings. For AF‐20% and AF‐40%, under low‐pressure conditions, the ability of CO_2_ to induce crystallization is limited, resulting in no significant change in T_g_. As the pressure increases, the T_g_ of the blend rises markedly by the effect of polymer crystallization, indicating a high sensitivity of T_g_ to pressure changes.

Figure 3h–j show the DSC melting curves, FTIR spectra, and XRD patterns of AF‐40% after 24 h of saturation under different CO_2_ pressures, respectively. As the CO_2_ pressure increases, the content of both α‐phase and β‐phase crystals in PVDF also increases, indicating a significant promoting effect of CO_2_ on crystal formation. Figure 3k illustrates the crystallinity at different pressures, showing an exponential increase with rising pressure. The increased CO_2_ absorption in the blend enhances the mobility of molecular segments, resulting in more perfect crystal formation and larger crystal sizes. Generally, there exists a CO_2_ saturation pressure threshold that can abruptly induce crystallization in the blend. With higher PVDF content, this pressure threshold decreases. In this system, crystallinity is influenced by both PVDF content and CO_2_ saturation pressure, with higher sensitivity observed under higher contents or pressures. The crystallinity and crystal size of the polymer significantly impact foaming, as these factors are key determinants of cell size and expansion ratio in the foam. Therefore, during the temperature rising foaming process, it is crucial to synergistically regulate the material's microwave absorption capability, solubility, crystallinity, and crystal size to prepare optimal foam.

### 2.3 Conduction‐Microwave Heating Assisted PMMA/PVDF Foaming

#### 2.3.1 Effect of Crystal Phase Morphology on Polymer Foaming

**Figure 4:**
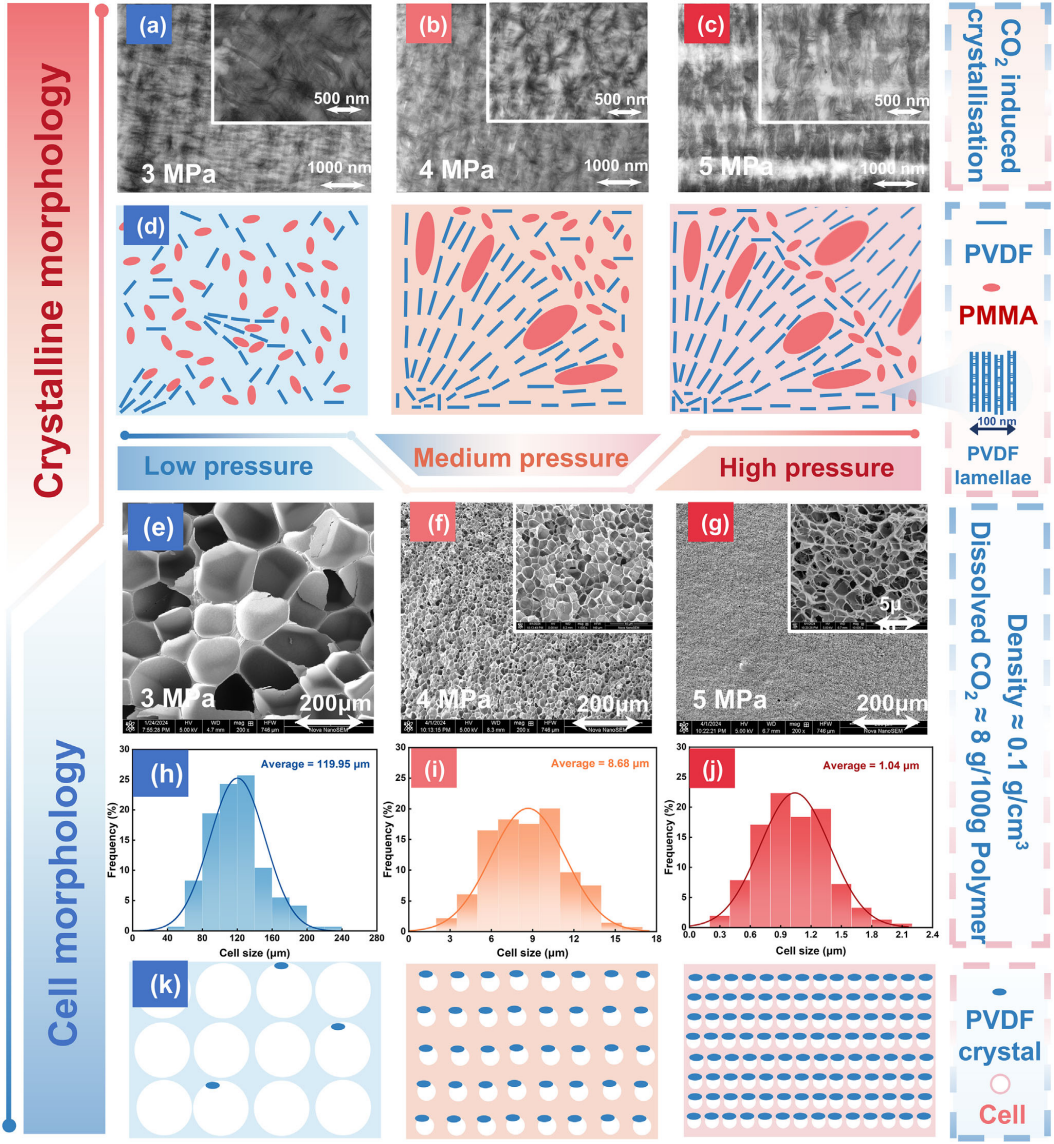
Crystallization Morphology of PVDF and Its Effect on PMMA/PVDF Foaming. Crystalline morphology (STEM images) of AF‐40% after saturation with CO_2_ for 24 h at a) 3 MPa, b) 4 MPa, and c) 5 MPa; d) Schematic diagram of CO_2_‐induced crystallization; Cell morphology (SEM images) after foaming to an expansion ratio of 15 following CO_2_ saturation at e) 3 MPa, f) 4 MPa, and g) 5 MPa; Cell size distribution after foaming to an expansion ratio of 15 following CO_2_ saturation at h) 3 MPa, i) 4 MPa, and j) 5 MPa; k) Schematic diagram of the influence of crystallization on cell morphology.

Under CO_2_ plasticization, polymers exhibit changes in their microstructure due to the evolution of crystalline phase morphology. **Figure** **4**a–c present TEM micrographs of AF‐40% after 24 h of saturation at different pressures. The results indicate the formation of microphase‐separated structures in the CO_2_‐treated PVDF/PMMA blends. This occurs because the enhanced mobility of molecular segments in the plasticized polymer drives similar polymer types to aggregate during movement. As plasticization intensifies, molecular segments tend to transition to a thermodynamically favorable crystalline state.

When the blend is treated at low temperature and under 3 MPa of low‐pressure CO_2_, the mobile PVDF molecular segments orderly aggregate to form lamellar crystals, forcing PMMA to be excluded. However, at low temperatures, the migration ability of the molecular chains is limited. The rates of PVDF crystal formation and PMMA exclusion are both slow, leading to the accumulation of PMMA at the ends of the lamellae, resulting in a “fluffy” crystal structure for PVDF. As the saturation pressure increases, more pronounced plasticization further enhances the mobility of molecular segments. Consequently, PVDF begins to form larger and more numerous crystals (Figure 4d).

The formation of PVDF crystals significantly affects the foaming behavior of the blend. As the temperature rises during the foaming process, PVDF microcrystals typically create numerous heterogeneous nucleation sites within the polymer matrix. This lowers the nucleation energy barrier, resulting in a significantly higher nucleation density of the foam cells, which is beneficial for producing foams with smaller cell sizes.^[^ ^10^ ^]^ Figure 4e–j show the microscopic structure of the cells and statistical results of AF‐40% foams expanded to ≈15 times after saturation under different pressures. Under low‐pressure saturation conditions, the few microcrystals present in the blend do not provide sufficient heterogeneous nucleation sites, leading to fewer nucleated cells and larger cell sizes dominated by homogeneous nucleation. As the saturation pressure increases, more numerous small microcrystals provide additional nucleation sites for the foam cells, significantly reducing the nucleation density. Furthermore, the increased crystallinity substantially enhances the rigidity of the matrix, offering significant resistance to cell growth and resulting in smaller cell sizes (Figure 4k). This suggests a viable approach for preparing high expansion ratio foams with small cell sizes. However, the significantly increased nucleation density limits the final expansion ratio of the foam. The rigid matrix formed by the microcrystals restricts cell growth, and the CO_2_ dissolved in the matrix is more consumed by cell nucleation, leaving insufficient CO_2_ for cell growth. Therefore, a comprehensive evaluation of the trade‐off between small cell size and high expansion ratio is essential to achieve high‐quality PMMA/PVDF foams.

#### 2.3.2 Regulation of PMMA/PVDF Foaming Effects

Solid‐state temperature rising foaming, a temperature‐induced foaming process, demands extremely uniform temperature distribution and precise temperature control. Traditional heating methods, such as liquid baths or hot air conduction, heat the material from the edges toward the core to achieve overall heating. However, for materials with significant thickness or low thermal conductivity, it takes considerable time to ensure uniform temperature distribution. This challenge is exacerbated when producing foams with high expansion ratios. During the foaming process, the increased size of the product hinders heat transfer, as the expanding foam creates microcells that reduce the material's thermal conductivity. Additionally, the rapid escape of CO_2_ from the matrix removes part of the heat. These combined factors result in low heat transfer efficiency, making it extremely challenging to produce foams with uniform internal cell structure and high expansion ratios.

**Figure 5:**
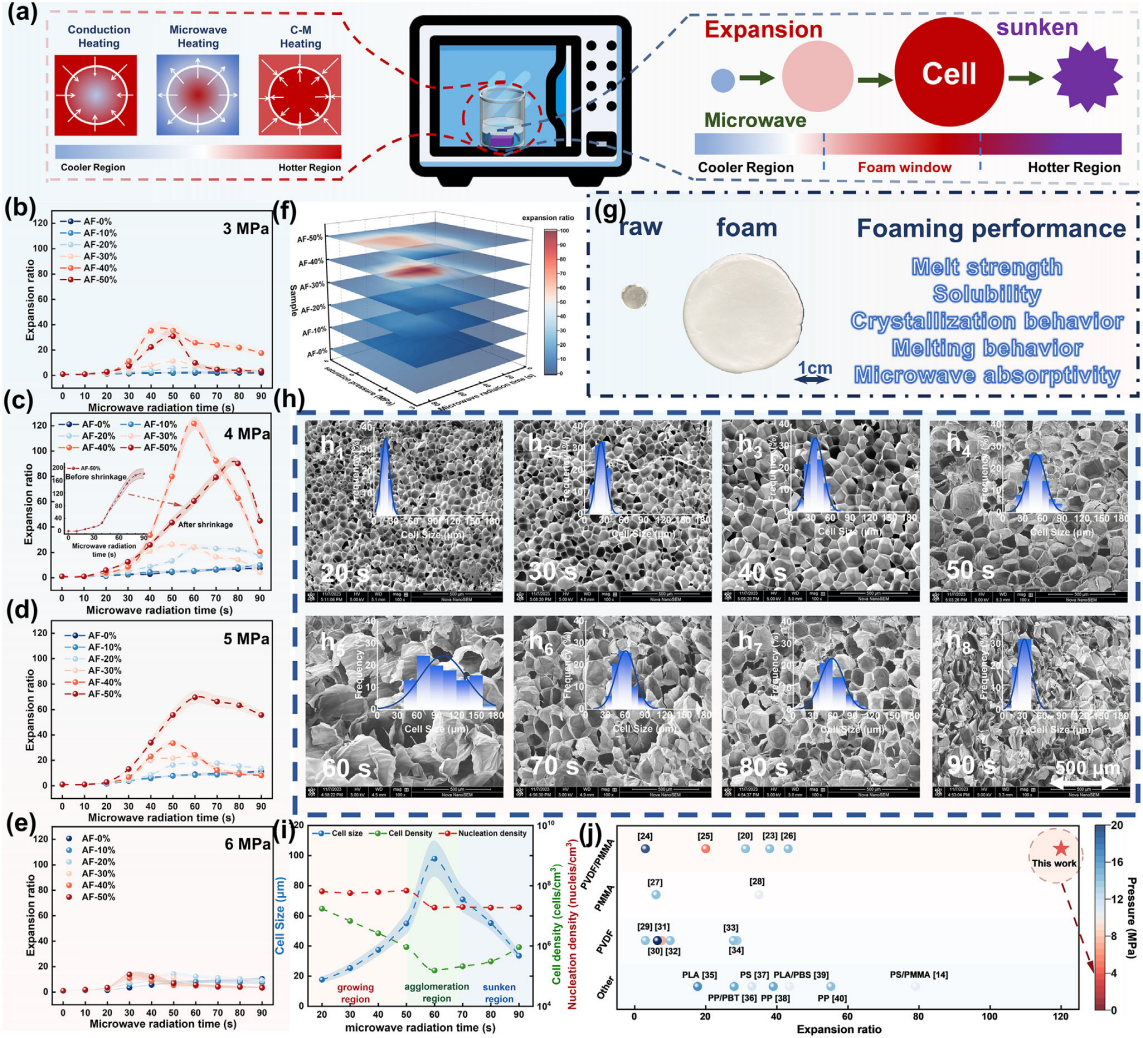
Macro and Microstructural Regulation of C‐M Heating Assisted Foaming. a) Schematic diagram of C‐M heating assisted foaming, showing uniform temperature distribution and foam expansion process; Changes in foam expansion ratios of PMMA and PMMA/PVDF after saturation with CO_2_ for 24 h at b) 3 MPa, c) 4 MPa, d) 5 MPa, and e) 6 MPa under 1000 W microwave radiation power for various microwave irradiation times; f) Changes in foam expansion ratios considering the combined factors of blend ratio, saturation pressure, and microwave irradiation time; g) Images of AF‐40% and AF‐40% foam with an expansion ratio of 120; h) Cell morphology and i) changes in average cell size, cell density, and nucleation density of AF‐40% after saturation with CO_2_ for 24 h at 4 MPa and heated by 1000 W microwave radiation for different times; j) Comparison of foaming pressure and expansion ratios of foams prepared in this study with similar materials in existing literature and foams claimed to have high expansion ratios.^[^ ^14^, ^20^, ^23^, ^24^, ^25^, ^26^, ^27^, ^28^, ^29^, ^30^, ^31^, ^32^, ^33^, ^34^, ^35^, ^36^, ^37^, ^38^, ^39^, ^40^ ^]^

Microwave‐assisted foaming is a promising solution to the above challenge. However, as shown by the temperature measurements in Figure 2a, heat exchange between the foam's edges and the cold air in the microwave apparatus during microwave foaming causes the edge temperature to be significantly lower than the center temperature. This results in the core region having a higher expansion ratio and larger cell sizes compared to the edge regions, severely impacting the uniformity of the foam cells. In our earlier research, we developed a conductive‐microwave (C‐M) hybrid heating mode to achieve efficient and uniform heating of the material (**Figure** **5**a). In this method, PVDF acts as an internal heating medium, absorbing microwave energy and converting it to heat for foaming. Simultaneously, water, used as an external heating medium, absorbs microwave energy to maintain the temperature at the edge of sample. By combining the advantages of conductive heating and microwave heating, the C‐M hybrid heating scheme enables simultaneous outside‐in and inside‐out heating, ensuring uniform foaming of PMMA/PVDF.

C‐M hybrid heating involves a continuous temperature rise process. With a fixed microwave power, the volume temperature of the heated material will continuously rise as the microwave radiation time extends. This results in foams with different morphologies under varying microwave radiation durations during the polymer foaming process. As depicted in Figure 5a, the initial polymer is a cold unfoamed sample after removal from the autoclave. The polymer matrix heats uniformly and rapidly as the microwave radiation time increases. When the temperature reaches the T_g_ of plasticized polymer, the mobility of molecular chains significantly increases, initiating the foaming process. Unlike traditional conductive heating methods, where the temperature remains constant during foaming, continuous heating in the C‐M hybrid approach causes the foam to expand further. However, when the foam is heated beyond its melting temperature, the internal cell structure risks collapsing, leading to foam shrinkage and a consequent reduction in expansion ratio.

Figure 5b–f illustrates the foaming effects of PMMA/PVDF under different saturation pressures following microwave irradiation. The results demonstrate that by adjusting the blend ratio, saturation conditions, and microwave irradiation parameters, the expansion ratio of the foam can be altered. However, the impact of these macroscopic variables on foaming ultimately stems from their influence on the microstructural properties. Figure 5g summarizes the key factors affecting the foaming performance of PMMA/PVDF, including melt strength (indicating whether the matrix exhibits rigidity or ductility), solubility, crystallization behavior (crystallinity, crystal size, and distribution), melting behavior (T_g_ and T_m_ post‐CO_2_ plasticization), and microwave absorption capability. These factors do not act independently but rather in a synergistic manner. Only by comprehensively considering all these factors can optimal foaming conditions be achieved.

According to Figure 5b–e, both excessively low and excessively high saturation pressures inhibit the foaming of PMMA/PVDF blends. As part of the foaming process facilitated by heating, a high concentration of dissolved foaming agent is essential for successful foaming. When the saturation pressure is too low, the amount of CO_2_ dissolved in the polymer matrix is insufficient. As the polymer expands, the limited amount of gas is rapidly consumed, leaving an inadequate quantity to sustain the growth of internal cells. Furthermore, since the C‐M hybrid heating‐assisted foaming is a continuous heating process, a broad foaming window is necessary to provide sufficient time for foaming under the given power conditions. However, lower saturation pressure results in weaker plasticization of the polymer. Consequently, PMMA/PVDF blends saturated at low pressure exhibit a higher T_g_ compared to those saturated at high pressure. Under the same microwave radiation power, it takes a longer time for these low‐pressure saturated blends to reach their foaming temperature. During this extended period, more CO_2_ escapes from the matrix, resulting in shorter foaming times and further lower dissolved foaming agent content compared to high‐pressure conditions, which limits expansion.

However, when the saturation pressure is excessively high, the formation of a high level of crystallinity content severely impedes cell growth, thereby affecting the expansion ratio. There are four main reasons for this. First, crystalline regions do not dissolve CO_2_ and cannot foam, relying solely on the amorphous regions for foaming. As the saturation pressure increases, both the size and number of crystals significantly increase, reducing the volume of the foamable regions. Second, the uniform and dense distribution of crystals within the polymer matrix enhances the rigidity of the polymer matrix. This increased melt strength obstructs the foaming of the amorphous regions. Third, the formation of crystals squeezes the CO_2_ dissolved in the polymer matrix, which leads to the outward expulsion of CO_2_, further reducing the amount of dissolved CO_2_. As a result, even at high saturation pressures, the solubility of CO_2_ in the polymer matrix does not improve appreciably. Fourthly, the microphase separation structure formed by crystallization raises the T_g_ of the polymer matrix. Since the polymer needs to reach its T_g_ to begin foaming, a higher T_g_ results in a shorter foaming window for the PMMA/PVDF blends. During continuous heating, excessive CO_2_ consumption and insufficient foaming time prevent the optimum expansion ratio from being achieved. Considering the overall effect of saturation pressure, only by selecting a moderate pressure (≈4 MPa) can ultra‐high expansion ratios exceeding 120 times.

The expansion ratio of PMMA/PVDF samples under different blend ratios exhibits significant differences when treated with identical saturation and microwave radiation conditions. First, samples with varying PVDF content show notable physical property differences even after identical treatments. Second, since the microwave absorption of material relies solely on PVDF, blends with low PVDF content exhibit weaker microwave absorption capability, making it challenging to achieve optimal foaming even after prolonged radiation. As depicted in Figure 5c, when the PVDF content is below 30%, the maximum expansion ratio reaches only 20 times. With the further increase in PVDF content, the expansion ratio significantly enhances, with a maximum expansion ratio of nearly 180 times at 50% PVDF content. Such high expansion ratios have not been reported in current literature, representing the highest expansion ratio achievable through CO_2_ foaming. However, excessive expansion results in very large cell sizes and extremely thin cell walls, making it difficult to maintain superior cell morphology. Consequently, the cells undergo rapid shrinkage due to thermal expansion and contraction after removal, resulting in a final expansion ratio of ≈90 times (with a porosity of 98.89%).

Figure 5h shows the cell morphology of AF‐40% after foaming at different microwave irradiation times following saturation under 4 MPa pressure, with cell data statistically analyzed in Figure 5i. The results demonstrate that the temperature variation induced by microwave irradiation plays a crucial role in the foaming process. For samples saturated under the same pressure and time, the expansion ratio initially increases and then decreases with prolonged microwave irradiation. At 20 s of irradiation, nucleation within the foam is essentially complete. As irradiation time extends, the matrix temperature gradually rises, causing the gas dissolved in the polymer matrix to diffuse into the gas nuclei and rapidly expand, leading to cell growth. However, at 60 s of irradiation, excessive expansion results in excessively thin cell walls. Concurrently, the matrix temperature reaches its optimal foaming limit, causing cell coalescence, which increases cell size and decreases nucleation density. With further irradiation, the polymer begins to melt, causing the cells to shrink, resulting in a gradual decrease in size until the cell walls become highly wrinkled, and the expansion ratio significantly decreases. Throughout this process, the cell growth can be aggregated into distinct stages: under 1000 W microwave irradiation, cells are in the growth stage for t ≤ 50s, in the aggregation stage for 50s < t < 70s, and in the rapid shrinkage stage for T ≥ 70s. For microwave‐assisted foaming systems, the expansion ratio largely depends on cell growth, reflecting similar trends in cell size changes. This provides a basis for predicting cell size under different irradiation conditions.

The expansion ratio (porosity) of cells is the most critical metric for assessing the quality of foam production. Figure 5j compares the expansion ratios of the foams prepared in this study with those reported in existing literature for the same system and other high expansion ratio foams.^[^ ^14^, ^20^, ^23^, ^24^, ^25^, ^26^, ^27^, ^28^, ^29^, ^30^, ^31^, ^32^, ^33^, ^34^, ^35^, ^36^, ^37^, ^38^, ^39^, ^40^ ^]^ The stabilized foams produced in this study exhibit a maximum expansion ratio exceeding 120 times (with a porosity of 99.17%). The superior expansion ratio is attributed to the exceptional crystallization control and microwave absorption characteristics of the PMMA/PVDF system, which align perfectly with microwave radiation. This expansion ratio significantly surpasses that of PMMA/PVDF foams produced by rapid depressurization foaming and traditional temperature rising foaming reported in the literature. Moreover, the ability to achieve such high expansion ratios under ultra‐low saturation pressure (only 4 MPa) demonstrates remarkable advantages in terms of energy and material savings, operational safety, and product quality.

### 2.4 Practicality of PMMA/PVDF Foams

**Figure 6:**
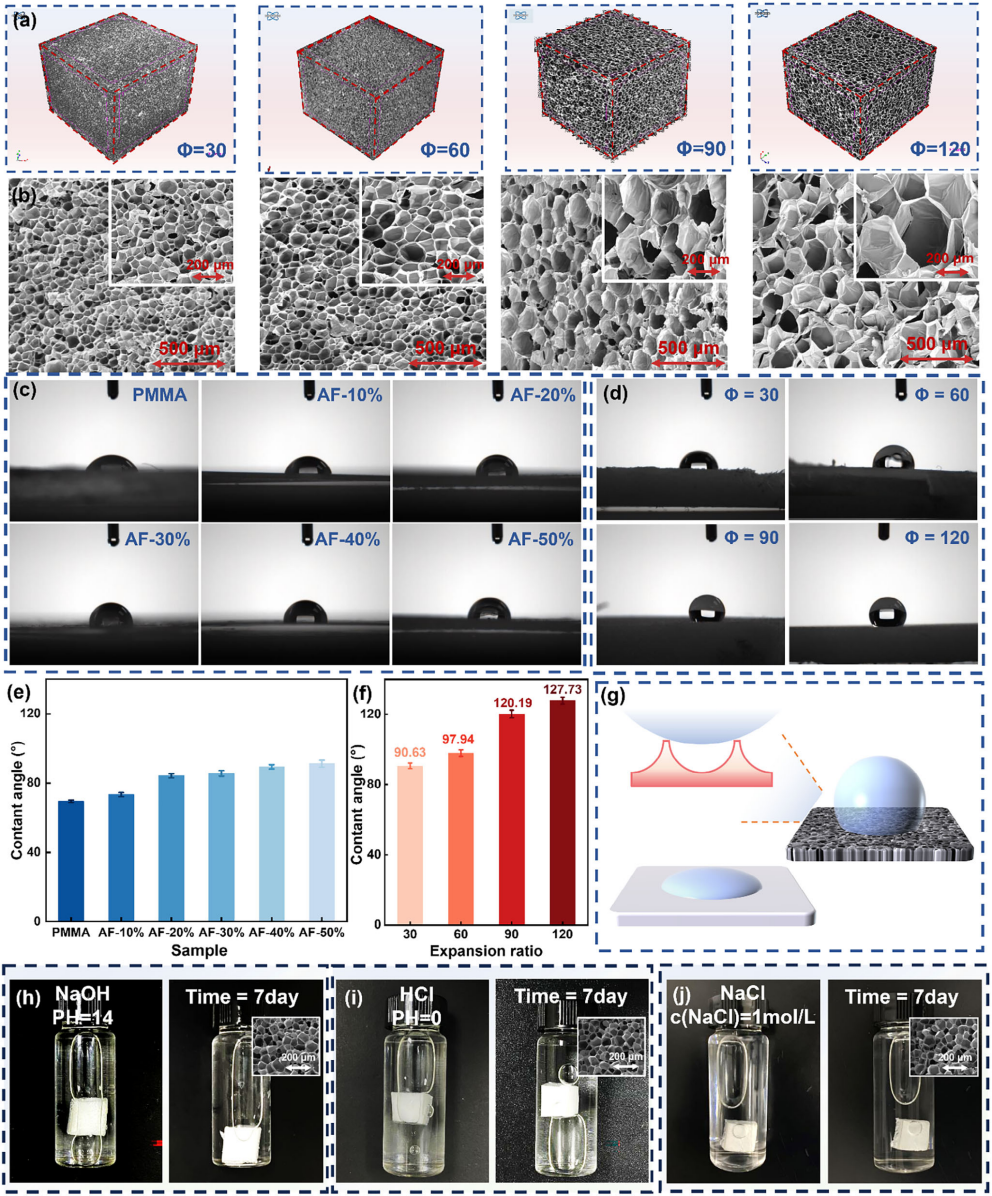
Practicality of PVDF/PMMA Foams. a) Micro‐CT and b) SEM of AF‐40% foams with expansion ratios of 30, 60, 90, and 120; c) Water contact angle images of PMMA and PMMA/PVDF; d) Water contact angle images of AF‐40% foams with expansion ratios of 30, 60, 90, and 120; e) Water contact angles corresponding to (c); f) Water contact angles corresponding to (d); g) Schematic of water contact angles on solid and foam surfaces; AF‐40% foam before and after immersion in h) NaOH solution (pH = 14), i) HCl solution (Ph = 0), and (g) 1 mol L^−1^ NaCl solution for 7 days.

The practicality metrics of the high‐expansion ratio PMMA/PVDF foams determine their potential for practical applications. Performance evaluations were conducted on AF‐40% foams with varying expansion ratios (*Φ* = 30, 60, 90, and 120, corresponding to porosities of 96.67%, 98.33%, 98.89%, and 99.17%, respectively). **Figure** **6**a,b display the microstructures of these four foams using macro‐CT and SEM, respectively. As analyzed in Section 2.3, the enhancement in foam expansion ratio largely depends on the significant growth in cell size. Therefore, the differences in foam expansion ratios show a synergistic trend with changes in cell size.

The water absorption of polymer foams seriously affects their performance, thereby reducing their lifespan as building materials. Typically, the hydrophobicity of foam materials is a crucial parameter for evaluating their practicality for outdoor use. Due to the differing affinities of PMMA and PVDF for water, polymer blends with varying PMMA/PVDF ratios exhibit inconsistent hydrophobicity. Figure 6c,e show the water contact angles of unfoamed PMMA and PMMA/PVDF. The abundance of hydrophilic ester groups in PMMA endows significant hydrophilicity. With increasing PVDF content, the introduction of fluorine groups lowers the surface energy of the polymer, leading to a transition toward hydrophobicity. The water contact angle of AF‐40% is ≈20° higher than that of PMMA. Figure 6d,f display the water contact angles of AF‐40% foams with different expansion ratios. The regular micro‐rough structures on the foam post‐foaming enhance its hydrophobicity^[^ ^41^ ^]^ (Figure 6g). The water contact angle increases with the cell size, reaching 127.73° at an expansion ratio of 120. Not only that, it also has a water absorption rate of only 0.00267 mol per 100g. The excellent hydrophobic properties of PMMA/PVDF foams ensure their surface exhibits waterproof, anti‐icing, anti‐fogging, and self‐cleaning characteristics during use.

Due to the influence of ester bonds, PMMA molecular chains are susceptible to degradation in acidic and alkaline environments,^[^ ^42^ ^]^ making it challenging to withstand the variable outdoor climate. However, the addition of PVDF dramatically improves the resistance to acids, bases, and salts. This improvement is attributed to two factors. First, the strong electronegativity of the fluorine element in PVDF endows excellent chemical stability by strongly repelling hydrogen bonds. Second, PVDF can spontaneously form a protective layer in acidic and alkaline environments, effectively preventing further internal corrosion. Figure 6h–j shows the condition of AF‐40% foam after being exposed to strong acid, strong base, and high salt environments for 7 days. Its degradation rates in all three environments tested were close to 0%. The results indicate that the foam maintained its structure, demonstrating superior chemical resistance compared to PMMA^[^ ^42^ ^]^ and meeting usage requirements even in extreme environments.

**Figure 7:**
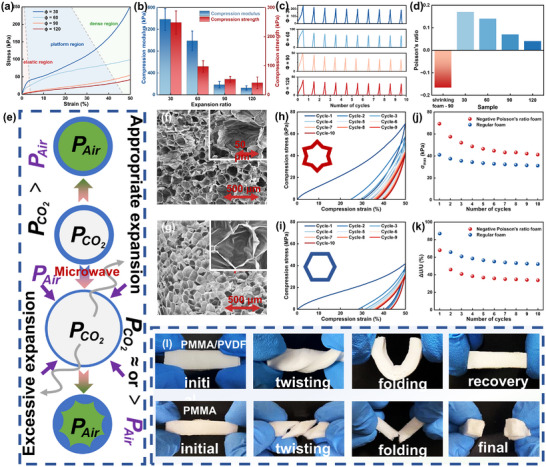
Mechanical Properties of PMMA/PVDF Foams. a) Compression stress‐strain curves; b) Compression strength and compression modulus; c) Stress variation over 10 compression cycles of AF‐40% foams with expansion ratios of 30, 60, 90, and 120; d) Poisson's ratio of AF‐40% foams with different expansion ratios compared with AF‐50% foam at an expansion ratio of 90 after shrinkage; e) Schematic diagram illustrating mechanisms for forming positive and negative Poisson's ratios in the foams; Cell morphology of f) AF‐40% foam expanded up to 90 times and g) AF‐50% foam shrunk up to 90 times; Strain‐stress curves of h) AF‐40% foam expanded up to 90 times and i) AF‐50% foam shrunk up to 90 times after 10 cycles of compression; j) Maximum stress and k) energy loss coefficient after 10 cycles of compression; l) Physical diagrams of PMMA/PVDF foam and PMMA foam after twisting and folding.

Robustness and resistance to compression are crucial factors for ensuring the long‐term, large‐scale outdoor use of foam materials. **Figure** **7**a shows the compression curves of AF‐40% foams with different expansion ratios. All foams exhibit similar compression behavior, undergoing elastic, plateau, and densification stages during compression. The data on compression strength and modulus are presented in Figure 7b. The results indicate that both compression strength and modulus decrease as the expansion ratio increases. This decline is attributed to the larger cell sizes and thinner cell walls at higher expansion ratios, which reduce the support capacity of the cell walls under the same strain. Nonetheless, the foam with an ultra‐high expansion ratio of 120 still demonstrates good compression strength, significantly outperforming materials with similar densities, such as aerogels, melamine foams, and EPS foams that are often claimed to be low‐density foams.^[^ ^43^, ^44^, ^45^ ^]^ Additionally, PMMA/PVDF foams exhibit excellent resilience. Figure 7c presents the compression curves for the four foams under 50% compression strain over 10 cyclic compressions. The results show that repeated compression cycles do not significantly affect the mechanical properties of the foam. This resilience is attributed to the incorporation of PVDF, which endows toughness to the material, thereby obviously enhancing the rebound performance of foam.

In particular, the AF‐50% foam, compressed to an expansion ratio of 90, exhibits unparalleled elasticity and compression performance compared to AF‐40% foam with the same expansion ratio. However, minor changes in content do not materially alter the properties of the material, and published data suggests that PMMA/PVDF with higher PMMA content typically exhibit better mechanical performance at the same expansion ratio,^[^ ^23^ ^]^ contrary to the results of this study. Figure 7d compares the Poisson's ratio of AF‐50% foam shrunk to an expansion ratio of 90 with AF‐40% foam at different expansion ratios. The results show that increasing the expansion ratio reduces the Poisson's ratio. The over‐expanded AF‐50% foam forms a negative Poisson's ratio structure when shrunken, with a Poisson's ratio of ‐0.17. Unlike normal materials, negative Poisson's ratio foams contract laterally during compression, resulting in high local packing density that resists compression forces.^[^ ^46^ ^]^

Figure 7e illustrates the formation mechanism of AF‐50% negative Poisson's ratio foam. The key to creating the concave and wrinkled cell walls lies in the combination of excessive expansion and rapid shrinkage of the foam.^[^ ^47^ ^]^ During appropriate expansion, CO_2_ dissolved in the polymer matrix diffuses into the cells, significantly increasing the internal pressure above the external environmental pressure, thus supporting the cells. However, with excessive expansion (an expansion ratio of up to 180 times), the internal pressure drops sharply due to the substantial utilization of CO_2_ for cell expansion. Additionally, the high PVDF content weakens the matrix rigidity, making the cell walls extremely thin and unable to support the cells. Upon removal from the water, the rapid cooling drastically reduces the internal pressure, causing air to exert an external force on the very thin cell walls, forcing them to shrink quickly. When the shrinkage speed is sufficiently fast, the cells fold inward to form concave structures rather than retracting uniformly to create thicker cell walls, ultimately resulting in a negative Poisson's ratio structure.

Figure 7f,g respectively demonstrate the cell structure differences between AF‐50% foam with an expansion ratio of 90 after shrinkage and AF‐40% foam with an expansion ratio of 90 without shrinkage. Although the two foams have similar cell sizes, their microstructures are quite different. The cell surface of the AF‐40% foam is relatively smooth, whereas the AF‐50% foam exhibits numerous wrinkled structures on the cell surface, ensuring a negative Poisson's ratio structure. Figure 7h,i present the compression curves for AF‐50% and AF‐40% foams, both with an expansion ratio of 90, under 50% compression strain over 10 cyclic compressions. The results show that the AF‐50% foam with a negative Poisson's ratio exhibits superior mechanical properties. The uniform distribution of compression forces across the ordered internal cell structure of foam ensures that the foam becomes denser and more resistant to compression as the cell walls come into contact with each other.

Figure 7j,k compare the maximum compression stress and energy loss coefficient of the two foams after 10 cyclic compressions. The results indicate that the foam with a negative Poisson's ratio structure exhibits a 69% increase in maximum compression stress and a 35% decrease in energy loss coefficient compared to conventional foam. This implies that incorporating a negative Poisson's ratio structure ensures that the foam is more compression‐resistant and resilient. Figure 6l compares the flexibility of PMMA/PVDF foam with PMMA foam. The PMMA/PVDF foam can be easily twisted or folded to any angle without damage, which is attributed to the addition of PVDF that transforms the material from rigidity to toughness. The foaming process further enhances the flexibility by introducing a cellular structure. The excellent mechanical properties, resilience to repeated compression, and flexibility of PMMA/PVDF foam ensure its adaptability to extreme outdoor conditions, meeting the needs of everyday use.

### 2.5 Innovative Applications of PMMA/PVDF Foams in Building Construction

**Figure 8:**
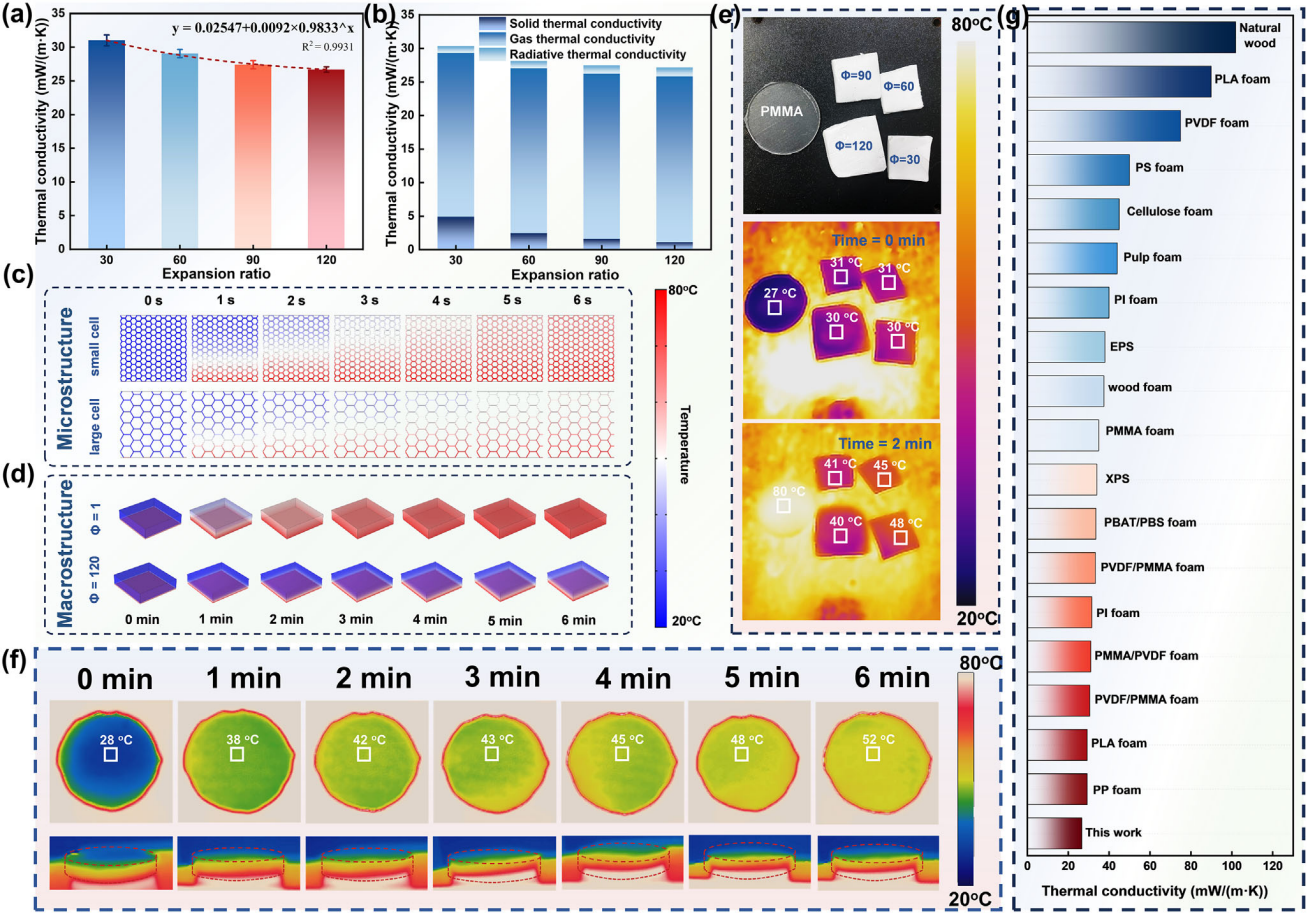
Thermal Insulation Properties of PMMA/PVDF Foams. a) Thermal conductivity of AF‐40% foams with different expansion ratios; b) Calculated solid‐phase, gas‐phase, and thermal radiation conductivities of AF‐40% foams with varying expansion ratios; c) COMSOL simulations illustrating the effect of different cell structures on foam insulation performance; d) COMSOL simulations illustrating the impact of various porosities on foam insulation performance; e) Infrared thermal images of PMMA and AF‐40% foams with different expansion ratios placed on an 80 °C heating plate for different durations; f) Infrared thermal images (top and side views) of AF‐40% foam with an expansion ratio of 90 over time on an 80 °C heating plate; g) Comparison of thermal conductivity between high expansion ratio foams prepared in this study, conventional commercial foams, and low thermal conductivity foams produced using CO_2_ foaming reported in the literature.^[^ ^20^, ^23^, ^26^, ^48^, ^49^, ^50^, ^51^, ^52^, ^53^, ^54^, ^55^, ^56^, ^57^, ^58^, ^59^, ^60^ ^]^

Modern building materials require to be kept warm in winter, while thermal insulation and passive radiant cooling are required in summer. High‐porosity foams, filled with air, typically exhibit excellent thermal insulation properties. **Figure** **8**a,b show the thermal conductivity measurements of AF‐40% foams with different expansion ratios, along with the calculated solid‐phase, gas‐phase, and radiative thermal conductivities. The presence of cells significantly reduces the thermal conductivity compared to unfoamed samples, with a reduction of approximately 85% at an expansion ratio of 30.^[^ ^20^ ^]^ This indicates that the air within the cells effectively impedes heat transfer. As the expansion ratio increases, the thermal conductivity of foam continues to decrease. Although larger cells at high expansion ratios contain more air, leading to a slight increase in gas‐phase thermal conductivity, the heat convection caused by weak gas exchange does not significantly impact overall thermal conductivity. However, the thin cell walls effectively disrupt the solid‐phase thermal conduction pathways, greatly reducing solid‐phase thermal conductivity, and thus the overall thermal conductivity. At an expansion ratio of 120, the gas‐phase thermal conductivity accounts for 92.5% of the total, with an overall thermal conductivity as low as 26.69 mW m^−1^ K^−1^), nearly approaching the thermal conductivity of air. This demonstrates that the foam has great potential for keep‐warm and thermal insulation.

Using multiphysics simulation software COMSOL, the thermal insulation properties of different foam structures and porosities in microwave‐assisted foaming systems were analyzed from both microscopic and macroscopic perspectives. For foams made from the same material, variations in expansion ratio primarily manifest in differences in cell size and cell wall thickness. Figure 8c compares the impact of structural differences between large cells (thin cell walls) and small cells (thick cell walls) on heat transfer. The results exhibit conclusions consistent with experimental and theoretical calculations that thinner cell walls can reduce the pathways for heat transfer and effectively impede solid‐phase heat transfer, resulting in better thermal insulation. When the foam with thick cell walls reaches thermal equilibrium, the foam with thin cell walls still shows a significant temperature gradient. Figure 8d analyzes the differences between foams and non‐porous solid materials by setting different porosities in the macroscopic structure. The formation of cell structures within the unit volume significantly enhances the thermal insulation of the material.

Figure 8e presents the heating of foams with different expansion ratios and the same thickness on an 80 °C hot plate. Within a 2 min heating period, the non‐porous polymer shows almost no insulation effect, whereas the foam creates a large temperature difference. As the expansion ratio increases, the temperature difference between the foam and the hot plate becomes more pronounced. Figure 8f shows the continuous heating of AF‐40% foam with an expansion ratio of 90 times at 80 °C. The top temperature of the foam gradually stabilizes at ≈50 °C, demonstrating excellent thermal insulation performance. Figure 8g ^[^ ^20^, ^23^, ^26^, ^48^, ^49^, ^50^, ^51^, ^52^, ^53^, ^54^, ^55^, ^56^, ^57^, ^58^, ^59^, ^60^ ^]^ compares our foam with existing conventional commercial foams and polymer foams with claimed low thermal conductivity from the literature.^[^ ^61^ ^]^ Our foam nearly achieves the lowest thermal conductivity possible for CO_2_‐foamed polymers, far outperforming conventional building insulation materials like expanded polystyrene (EPS) and extruded polystyrene (XPS), showcasing significant advantages and potential as a perfect replacement in the field of building insulation and thermal materials.

**Figure 9:**
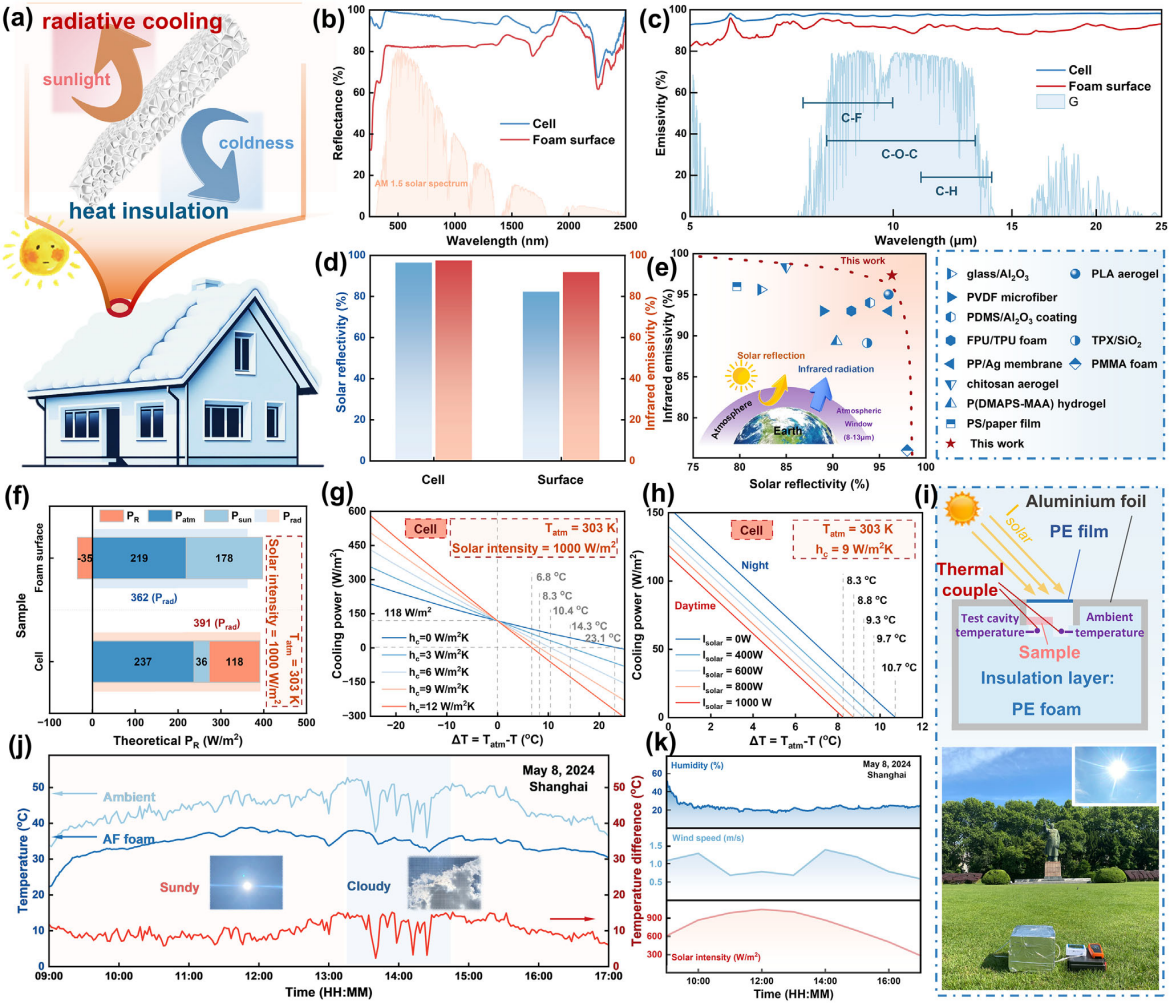
Passive Daytime Radiative Cooling Performance of PMMA/PVDF Foams. a) Schematic illustration of foam with thermal insulation and passive daytime radiative cooling capabilities applied in building construction; b) Solar reflectance of foams with skin surface and cell surface in the 250–2500 nm spectral range; c) Infrared emissivity of foams with skin surface and cell surface in the 5–25 µm spectral range; d) Average solar reflectance and average infrared emissivity of foams with skin surface and cell surface; e) Comparison of solar reflectance and infrared emissivity between high‐expansion ratio foams from this study and radiative cooling materials from existing literature^[^ ^8^, ^63^, ^64^, ^65^, ^66^, ^67^, ^68^, ^69^, ^70^, ^71^, ^72^ ^]^; f) Theoretical power density of thermal radiation (P_rad_), absorbed atmospheric radiation (P_atm_), absorbed solar radiation (P_sun_), and net radiative cooling power (P_R_) for foams with skin and cell surfaces; g) Variation of theoretical cooling power with temperature difference for foams with cell surfaces at different heat transfer coefficients; h) Variation of theoretical cooling power with temperature difference for foams with cell surfaces at different solar intensity; i) Schematic and actual setup of the test box used to characterize radiative cooling power and cooling temperature; j) Temperature data and temperature difference from outdoor tests conducted in Shanghai on May 8, 2024; k) Real‐time data of relative humidity, wind speed, and solar intensity.

Materials that combine thermal insulation with passive daytime radiative cooling can reduce direct solar heating while preventing outdoor temperatures from affecting the indoor environment. This effectively mitigates the impact of external light and temperature on indoor cooling systems, providing a viable solution for energy conservation and sustainable building design (**Figure** **9**a). Polymers with low extinction coefficients in the solar spectrum are ideal for radiative cooling. Specifically, PVDF and PMMA, with their internal chemical bond stretching vibrations, reduce solar intensity attenuation during light transmission, leading to low solar absorption.^[^ ^4^, ^62^ ^]^ Moreover, inspired by biomimicry from nature, introducing porous structures on the material surface is an effective way to enhance solar reflection or scattering because of the mismatch in refractive indices between the structure and air. Figure 9b depicts the reflectance spectra of the surface porous structure (cell) and foam skin layer (foam surface, Figure S7, Supporting Information) of AF‐40% foam in the UV‐visible‐NIR wavelength range. The skin layer without substantial micro‐nano structures achieves an average solar reflectance (${\bar{R}}_{solar}$) of 82.22% due to the inherent optical properties of the polymer. However, the introduction of surface porous structures significantly enhances ${\bar{R}}_{solar}$, reaching 96.37%. This indicates that the synergistic effect of the intrinsic optical properties of the material and the enhanced optics of the surface structures effectively reflect solar radiation and mitigate solar heating.

Another requirement for passive daytime radiative cooling is that the material exhibits high emissivity within the atmospheric window, effectively radiating excess energy into cold outside space. Figure 9c shows the emissivity of the foam within the atmospheric transmission window. PMMA and PVDF contain abundant chemical bonds such as C‐O‐C, C‐F, and C‐H, which undergo bending or stretching vibrations in the spectral range of 4000–400 cm^−1^. These vibrations resonate with broad infrared wavelengths, resulting in strong absorption. Enhanced by the surface porous structure, the long‐wave infrared emissivity (${\bar{\varepsilon }}_{LWIR}$) reaches up to 97.34% (Figure 9d). As shown in Figure 9e, compared with existing literature, our PMMA/PVDF foam exhibits both high solar reflectance and high infrared emissivity within the atmospheric window, demonstrating considerable potential for application in passive daytime radiative cooling.^[^ ^8^, ^63^, ^64^, ^65^, ^66^, ^67^, ^68^, ^69^, ^70^, ^71^, ^72^ ^]^

To quantify the cooling effect of the radiative cooler, its theoretical radiative cooling power was calculated, assuming an ambient temperature of 303 K. Figure 9f illustrates the impact of surface porous structures on the theoretical radiative cooling power. The daytime net radiative cooling power (*P_R_*) of the foam with a skin layer is less than zero, while the foam with a porous surface achieves a P_R_ of 118 W m^−2^, demonstrating excellent radiative cooling potential. As shown in Figure 9g, when considering a solar intensity of 1000 W m^−2^ and heat transfer coefficients (*h_c_*) of 0, 3, 6, 9, and 12 W m^−2^ K^−1^, the corresponding ideal cooling temperatures are 23.1, 14.3, 10.4, 8.3, and 6.8 °C, respectively, indicating ideal cooling performance. Meanwhile, the results indicate that strong solar irradiance can suppress the cooling performance of the radiative cooler (Figure 9h). Adverse environmental factors can significantly hinder its cooling performance in outdoor tests. However, when h_c_ is 9 W m^−2^ K^−1^, the cooling temperature under 1000 W m^−2^ solar intensity is only ≈2.4 °C lower than that without solar radiation, suggesting that its radiative cooling capability is not significantly sensitive to changes in solar intensity.

The actual radiative cooling performance may differ from the theoretical results due to weather variations or differences in the use of the radiative cooler. Therefore, we selected a day with favorable weather conditions (May 8, 2024) to conduct outdoor solar testing of the radiative cooler in Shanghai, China (31.14° N, 121.42° E). The radiative cooler was placed inside a low‐density polyethylene foam brick with internal grooves, sealed at the top with a polyethylene film to minimize heat transfer and convection effects during the test. Additionally, the foam brick was wrapped in aluminum foil to eliminate the impact of direct sunlight on the test box (Figure 9i). The outdoor temperature measurement results shown in Figure 9j indicate significant fluctuations in ambient temperature, while the test cavity temperature changed more gradually and exhibited a delay compared to the ambient temperature. This is because the radiative cooler has excellent insulation properties, which reduce the influence of external environmental conditions on the temperature of the test cavity, making the internal temperature less sensitive to environmental changes (Figure 9k).

The temperature difference between the test cavity protected by the radiative cooler and the ambient temperature was considerable. When the ambient temperature reached 50 °C, the test cavity temperature was only 35 °C, resulting in a temperature difference of ≈15 °C. The actual radiative cooling power was ≈104 W m^−2^, closely matching the theoretical calculation results (118 W m^−2^). Both theoretical calculations and real‐time temperature measurements indicate that the radiative cooler, with its low thermal conductivity, high solar reflectance, and high infrared emissivity, demonstrates excellent daytime passive radiative cooling performance, offering potential applications for building insulation and cooling.

**Figure 10:**
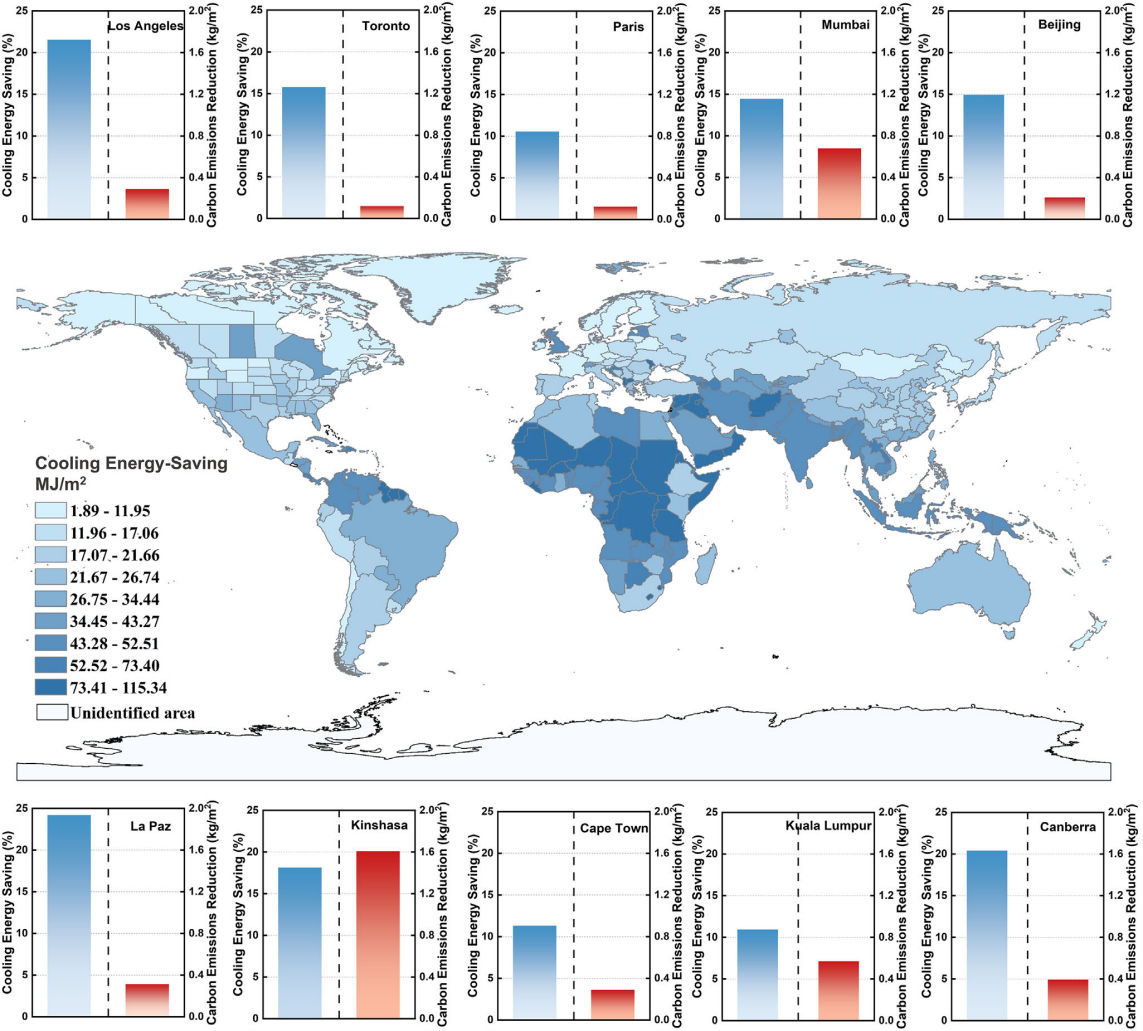
Global Cooling Energy Savings and Carbon Emission Reduction for Key Cities (Los Angeles, Toronto, Paris, Mumbai, Shanghai, La Paz, Kinshasa, Cape Town, Kuala Lumpur, and Canberra) Potential Forecast.

The passive radiative cooling in summer and thermal insulation in winter provided by PMMA/PVDF foam significantly contribute to energy savings and carbon emission reduction. Utilizing EnergyPlus, we simulated the energy savings of a four‐story apartment building (with a floor area of 782 m^2^) before and after the application of PMMA/PVDF foam as the building enclosure (including wall panels and roofs) to assess the energy savings and carbon emission reduction potential of the radiative coolers. During the energy consumption simulation, the indoor boundary conditions were maintained at a constant temperature of 26 °C, while the outdoor boundary conditions were derived from typical meteorological year hourly weather data. The control equations were solved iteratively with a 1‐hour time step over the course of a year to predict the energy‐saving and carbon emission reduction behavior of the radiative coolers across various countries and regions globally (**Figure** **10**). The results indicate that cities with hot and arid climates near the equator exhibit superior energy savings potential, with the highest cooling energy savings in Central Africa exceeding 110 MJ m^−2^. Compared to conventional building enclosure, these regions demonstrated over 20% reductions in energy consumption, effectively alleviating the energy load on active indoor cooling systems. Additionally, regions within the Tropics of Cancer and Capricorn also exhibit remarkable carbon emission reduction capabilities, with maximum reductions exceeding 1.5 kg m^−2^. This substantial decrease in energy demand for air conditioning systems underscores the effectiveness of this technology in promoting sustainable development and offers a viable pathway and feasible solution for achieving carbon neutrality in the global energy system.

## 3 Conclusions

We utilized a C‐M heating‐assisted foaming process to produce PMMA/PVDF foams with ultra‐high expansion ratios, leveraging the inherent optical properties and designed structural characteristics of the material for innovative applications in the construction industry. By adjusting foaming process parameters such as blend ratio, saturation time, and saturation pressure, we controlled the crystallinity and crystal morphology of the blend as well as its solubility in CO₂. The combined optimization of material properties and microwave processing resulted in the production of uniform foams with ultra‐high expansion ratios (exceeding 120 times) of PMMA/PVDF. The inherent properties of the material and the design of the pore structure endow the prepared foams with a low thermal conductivity (26.69 mW m^−1^ K^−1^), high solar reflectance (96.37%), and high infrared emissivity (97.34%). These features enable the foams to provide keep‐warm during winter and passive cooling of building exteriors as well as thermal insulation of interiors during summer. In theoretical radiative power calculations and practical application demonstrations, the radiative cooling power of our foams was 118 W m^−2^ and 104 W m^−2^, respectively, demonstrating significant energy‐saving potential. Furthermore, the high expansion ratio radiative cooling foams also exhibit hydrophobicity, chemical resistance, and recyclability. The rapid contraction‐induced negative Poisson's ratio structure endows high compression strength, elasticity, and excellent flexibility, rendering the foams suitable for both conventional climates and extreme weather conditions. These foams can be easily applied on a large scale to various scenarios and outdoor infrastructure. With an energy‐efficient, eco‐friendly, and green foam preparation process, our radiative cooling foams show immense potential in the field of next‐generation innovative building design, providing solutions for developing more sustainable and energy‐efficient buildings in the future.

## 4 Experimental Section

### Materials

PMMA (CM‐207, density 1.19 g cm^−3^) was purchased from Chi Mei Corporation. PVDF (Kynar Flex 2850‐04, density 1.77 g cm^−3^) was obtained from Arkema Group. CO₂ (99.995%) and N₂ (99.995%) were supplied by Air Production Co., Ltd. (Shanghai, China).

### Preparation of PMMA/PVDF Composites

The drying of PMMA and PVDF was conducted in a vacuum oven (DZG‐6050, Senxin Laboratory Instrument Co., Ltd., China) at 80 °C for 24 h in order to completely remove moisture. Subsequently, the dried polymers were blended in different ratios at 200 °C using a torque rheometer (Haake Polylab 16OS, Thermo Fisher Scientific, USA) at a rotation speed of 60 rpm for 10 min. The blended composites were subjected to hot pressing into discs with a diameter of 10 mm and a thickness of 2 mm. This was achieved using a hot press (XTC101B, Xintai Cheng Machinery Equipment Co., Ltd., China) at 200 °C under a pressure of 10 MPa. The PMMA/PVDF composites were designated as AF‐x, where x represents the mass content of PVDF.

### Preparation of PMMA/PVDF Foams

The samples were placed in a custom‐made high‐pressure autoclave with a volume of 250 mL. The autoclave was purged three times with CO_2_ to ensure the complete removal of air. CO_2_ was introduced into the autoclave at room temperature using an ISCO pump (100DX, Teledyne ISCO, USA) at a pressure range of 3–6 MPa. After saturation for 24 h, the pressure was slowly released at a rate of 0.5 MPa min^−1^ until the autoclave was completely depressurized. Following previous work,^[^ ^16^ ^]^ the saturated samples were placed in a microwave‐transparent container filled with 40 ml of water (temperature of 25 °C). A microwave‐transparent PTFE porous baffle was placed above the samples using a support to ensure the samples were fully immersed in water. The container holding the samples and baffle was then subjected to microwave radiation at 1000 W in a microwave oven (NN‐GF39JS, Panasonic Corporation, Japan, microwave frequency is 2450 MHz, output power is 1000 W) for specific times ranging from 0 to 90 s, achieving foaming through a combined conduction‐microwave heating method. The foamed samples were immediately placed in an ice‐water bath at 0 °C to halt further expansion.

### Computational Simulation and Material Characterization

Computational simulations (including Density Functional Theory and COMSOL) and processes of various testing and characterization methods (including characterization of raw material properties and the structure and performance of the foams) are detailed in the Supporting Information.

## Conflict of Interest

The authors declare no conflict of interest.

## Author Contributions

W.‐Y.Z. and Y.‐C.C. contributed equally to this work. L.Z. obtained the idea, designed research, and supervised the project. W.‐Y.Z. and Y.‐C.C. prepared the samples. W.‐Y.Z., D.‐D.H., and J.‐Y.S. characterized the samples and tested their performance. W.‐Y.Z. and J.‐Y.S. performed the simulations and validations. L.Z., W.‐Y.Z., and Y.‐C.C. prepared the manuscript. All authors commented on the manuscript.

## Supporting information



Supporting Information

## Data Availability

The data that support the findings of this study are available from the corresponding author upon reasonable request.
