# Supplementary material for: Unprecedented Ultra‐High Expansion Ratio Foam for Innovative Architecture
Source: Adv Sci (Weinh). 2025 Mar 24;12(19):2501188. doi: 10.1002/advs.202501188 (PMC12097086; doi:10.1002/advs.202501188)
Supplement: Supplementary file 1 — Supporting Information [file ADVS-12-2501188-s001.docx]

**Supporting Information**

**Unprecedented** **Ultra-high Expansion Ratio Foam for Innovative Architecture**

**Wenyu Zhong^a, 1^, Yichong Chen^a, b, 1^, Dongdong Hu^a^, Jiayang Sun^a^, Xingyu Jia^a^, Ling Zhao^a, b, *^**

1. State Key Laboratory of Chemical Engineering, Shanghai Key Laboratory of Multiphase Materials Chemical Engineering, School of Chemical Engineering, East China University of Science and Technology, Shanghai 200237, P. R. China
2. Shanghai Electronic Chemicals Innovation Institute, East China University of science and Technology, Shanghai 201419, P. R. China

*Corresponding author: Tel.: +86 21 64253175; Fax: +86 21 64253528

*E-mail: zhaoling@ecust.edu.cn (L. Zhao)

1: These authors contributed equally: Wenyu Zhong, Yichong Chen

**1 Simulation**

**1.1 Density Functional Theory (DFT)**

The optimization of the molecular geometry of VDF and MMA was conducted using DFT with the Gaussian 09 program package. Quantum chemical calculations were performed using the B3LYP/6-311G method. During the process, structure optimization and frequency calculations were carried out to ensure that all molecular structures attained their lowest energy state within the potential energy landscape. Additionally, electrostatic potential (ESP) maps were generated using the Multiwfn program to investigate the charge distribution of the molecules.

**1.2 COMSOL Simulation**

The thermal insulation properties of different foam cell structures and porosities were analyzed using the multiphysics simulation software COMSOL. The thermal conductivity of the polymer matrix was set to 205 mW/(m·K)^1^, and the thermal conductivity of air was set to 26 mW/(m·K) [2], with other parameters based on the built-in settings of the software. The size parameters for large and small cells are shown in **Figure S1**.


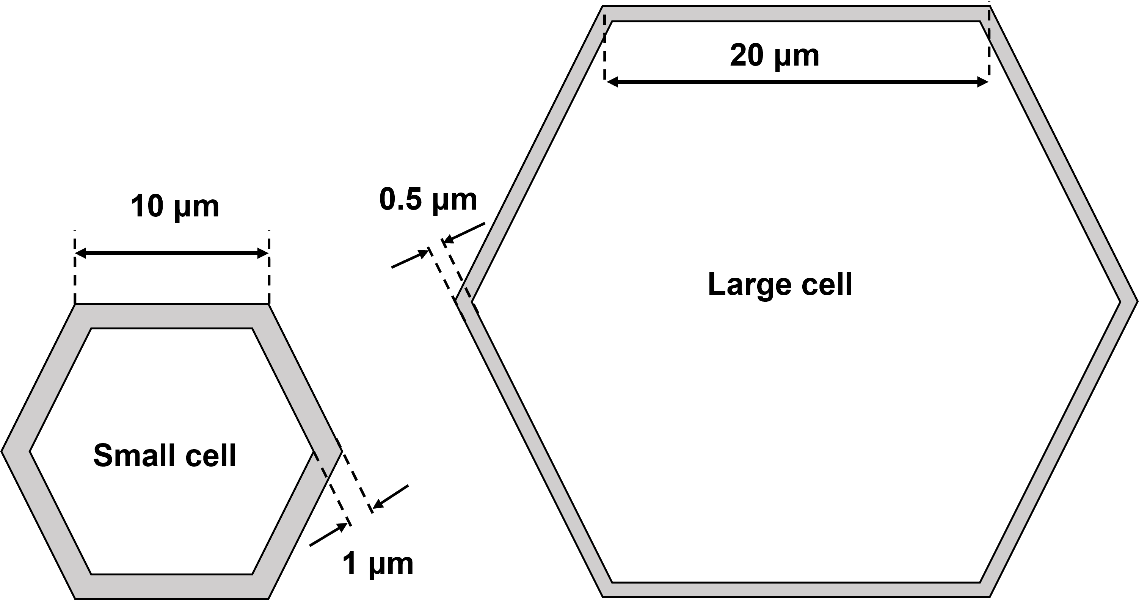


**Fig. S1 Cell Models and Parameters for COMSOL Simulations.**

During this simulation, the electromagnetic wave model (**Equation (S1)**) and the fluid-solid heat transfer (**Equation (S2)**) modules are solved simultaneously by using COMSOL's built-in physical coupling.

| $\nabla\times\frac{1}{\mu^{'}}\left( \nabla\times\vec{E} \right)-k_{0}^{2}\left( \varepsilon_{r}-\frac{j\sigma}{\omega\varepsilon_{0}} \right)\vec{E}=0$ | (S1) |
| --- | --- |
| $\rho C_{P}u\times\nabla T+\nabla q=Q\in q=-k\nabla T$ | (S2) |

In the electromagnetic wave module, the boundary condition of the input port can be assessed in **Equation (S3)**.

| $S=\frac{\int_{\partial\Omega} \left( E-E1 \right)\cdot E1}{\int_{\partial\Omega} E1\cdot E1}$ | (S3) |
| --- | --- |

Where *μ'* is the relative permeability, $\vec{E}$ is the electric field vector, *k_0_* is the wave number in vacuum, *ε_0_* is the permittivity in free space, *ε_r_* is the permittivity of the material, *j* is the imaginary unit, σ is the electrical conductivity, *ω* is the angular frequency, *S* is the scattering parameter, ρ is the density of the heated material, *C_p_* is the specific heat capacity at constant pressure, *u* represents the velocity field, *k* is the thermal conductivity, *T* is the temperature. *Q* represents the heat source or sink, which can include one or several heat sources as separate physics features. Additionally, *q* represents the heat flux and describes Fourier's law of heat conduction. The domain of materials in the model is divided by a normal size tetrahedral mesh, which is fine enough for model computation and can ensure good operation speed.

**1.3 Energy Saving Performance Simulation**

The energy saving simulation was conducted using EnergyPlus. The building energy savings of the specimens were estimated from a whole building energy simulation. A typical 4-story mid-rise apartment building was used as the prototype, with a floor dimension of 46 m (152 ft., L) × 17 m (55.5 ft., W) × 3 m (10 ft., H). This prototype building was developed by the Pacific Northwest National Laboratory of the U.S. Department of Energy (**Figure S2**). The model follows applicable building codes and standards for energy efficiency requirements, adopted typical operation and occupancy schedule, and defined typical lighting/miscellaneous loads and HVAC systems. Building energy performance is obtained by measuring the difference in values between the presence and absence of the addition of our radiant coolers. In the simulation, the specimens are applied as the outer layer of the external wall and roof.


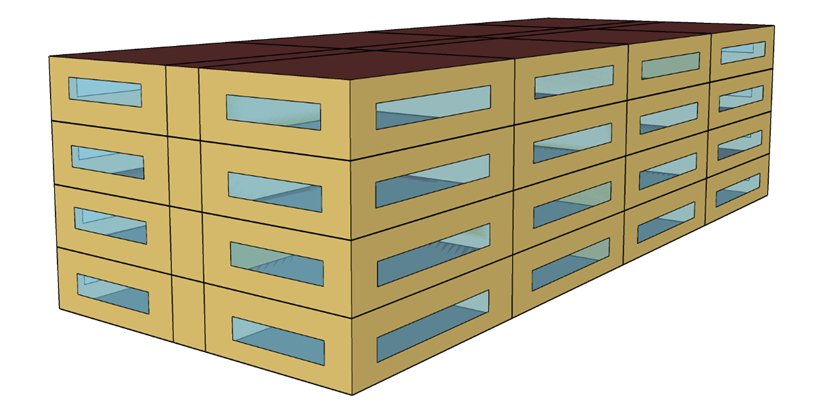


**Fig. S2 Building Modelling for Energy Saving Performance Simulation.**

**
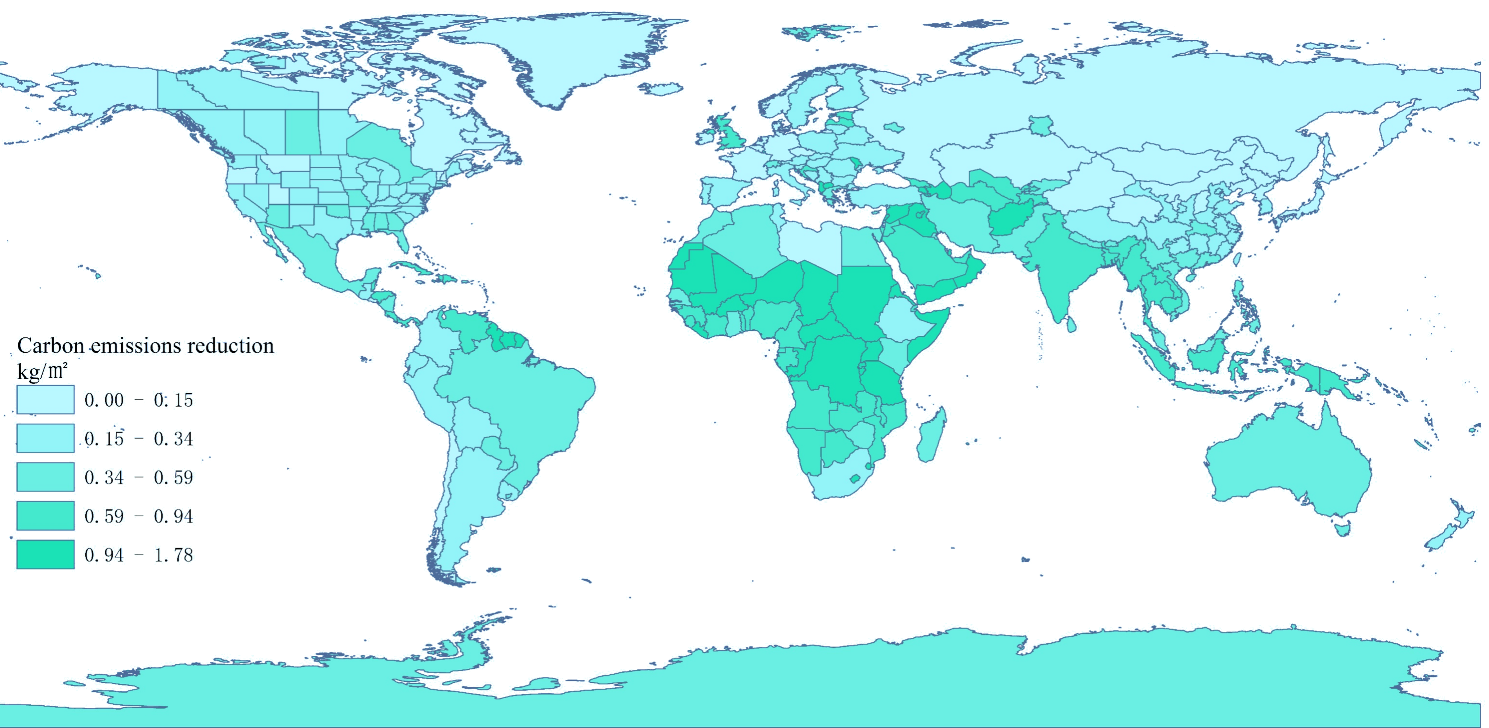
**

**Fig. S3 Global Carbon Emission Reduction Potential Forecast.**

**2 Characterize of** **Raw Materials**

**2.1 Dissolved Quantity**

The dissolved quantity of PMMA and PMMA/PVDF in CO_2_ environment ware assessed through gravimetric method. A designated mass of sample (denoted as *m_1_*) was introduced into an autoclave, which was purged with CO_2_ to ensure the complete removal of atmospheric air. Subsequently, CO_2_ was introduced into the autoclave at ambient room temperature (26 ± 2 °C), under varying pressures of 3-6 MPa. Following specific durations of saturation, the CO_2_ was slowly released (0.5 MPa/min) from the autoclave. After each saturation period, the sample was promptly extracted from the autoclave and weighed (denoted as *m_2_*) meticulously using an analytical balance. It is acknowledged that depressurization and gravimetric measurements conducted over short time intervals may introduce some degree of gas loss. It is noteworthy, however, that these losses exhibit a synchronized pattern during each measurement. This synchronization implies that it is reasonable and justifiable to discern alterations in the dissolved quantity of CO_2_ throughout the experimental procedure. The dissolved quantity (*D_q_*) can be determined by **Equation (S4)**:

| $D_{q}=\frac{m_{2}-m_{1}}{m_{1}}\times100\%$ | (S4) |
| --- | --- |

**2.2 Crystallinity and Crystal Morphology**

Differential Scanning Calorimetry (DSC) (NETZSCH DSC 204HP, Germany) was used to evaluate the melting behavior of PMMA and PMMA/PVDF under a nitrogen (N_2_) atmosphere. The test procedure for PMMA and PMMA/PVDF was as follows: 5 to 10 mg of the sample was placed in a crucible. All samples were heated from 40 °C to 180 °C at a rate of 10 °C/min, then held at 180 °C for 5 min, and finally cooled to 40 °C at a cooling rate of 10 °C/min. Finally, the samples were heated from 40 °C to 180 °C at a rate of 10 °C/min to analyze the differences in melting behavior among various samples.

To measure the impact of CO_2_ on the crystallization of composite materials, samples saturated under different pressures for 24 hours were first removed from the autoclave. These samples were then stored at -40 °C in a freezer for one month to prevent foaming. Afterward, they were kept at room temperature for an additional month to ensure complete gas escape. The test procedure for saturated PMMA and PMMA/PVDF was as follows: 5 to 10 mg of the sample was placed in a crucible. All samples were heated from -70 °C to 180 °C at a rate of 10 °C/min to analyze the differences in melting behavior among various samples. The crystallinity of the blends was calculated according to **Equation (S5)**:

| $X_{c,DSC}=\frac{\Delta H_{m}-\Delta H_{c}}{\varphi\Delta H_{m}^{0}}\times100\%$ | (S5) |
| --- | --- |

where $\Delta H_{m}$ is the melting enthalpy, $\Delta H_{c}$ is the cold crystallization enthalpy, $\varphi$ is the quality content of PMMA, $\Delta H_{m}^{0}$ is the melting enthalpy of the fully crystalline PVDF (104.76 J/g) [2].

Once the gas had completely escaped from the saturated PMMA and PMMA/PVDF samples, their crystallization states were analyzed using X-Ray Diffraction (XRD, D8 Advance, Germany). The samples were scanned within the 10-50 ° range at a rate of 2 °/min.

After being hot-pressed into thin films, the PMMA and PMMA/PVDF samples were saturated under different CO_2_ pressures. Once the gas had fully escaped, Fourier Transform Infrared Spectroscopy (FTIR, Spectrum 100, USA) was employed to scan the samples within the 600-1500 cm^-1^ spectral range, at a resolution of 2 cm^-1^ and 64 scans.

The AF-50% samples were hot-pressed into thin films and saturated with CO_2_ at 4 MPa. After complete gas escape, the crystalline morphology was captured using a Polarized Optical Microscope (POM, LAICA DM 2700 P, Germany).

Scanning transmission electron microscopy (STEM, MultiView 4000, Nanonics Imaging Ltd., German) was used to characterise the microstructure of the crystals. For this analysis, sections of the samples were cut at room temperature into thin films of 90 nm thick on a Leica EM UC6/FC6 ultra-microtome and placed on 200 mesh copper grids.

**2.3 Rheological Behavior**

The dynamic oscillatory shear rheological behaviors of PMMA and PMMA/PVDF were investigated using a HAAKE MARS III rotational rheometer equipped with a 35 mm disk and a 1.8 mm gap (Thermo Fisher Scientific Inc. Co., USA). The measurements were carried out under a N_2_ atmosphere at the specified measurement temperature to prevent oxidative degradation. Prior to testing, all samples underwent vacuum drying at 80 °C for 24 h to eliminate volatile residues.


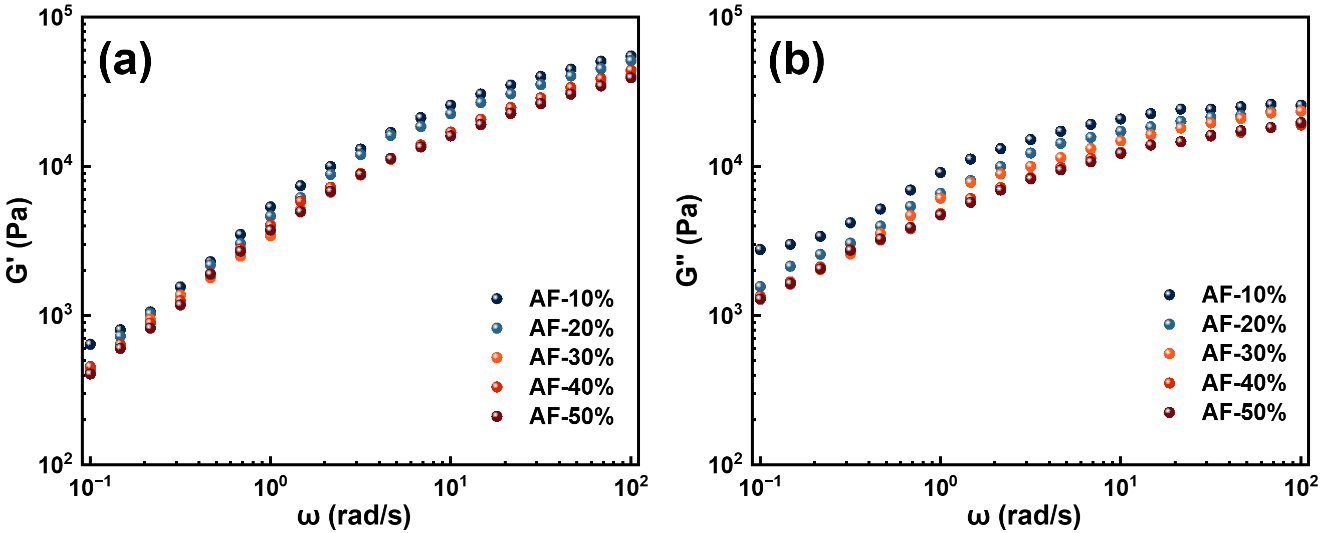


**Fig. S4 Variation of (a) Energy Storage Modulus and (b) Loss Modulus of PMMA/PVDF with Angular Frequency.**

PMMA and PMMA/PVDF sheets, each with a diameter of 35 mm and a thickness of 2 mm, were manufactured using an injection molding machine (Wuhan Ruiming Experimental Instrument Co., Ltd., China) at a temperature of 190 °C. Frequency sweeps were conducted in the range of 0.1 to 100 rad/s within the linear viscoelastic region at 190°C. During the rheological measurement, the stress value for the oscillation frequency sweep was maintained at 10 Pa to ensure a linear viscoelasticity region for both PMMA and PMMA/PVDF.

**
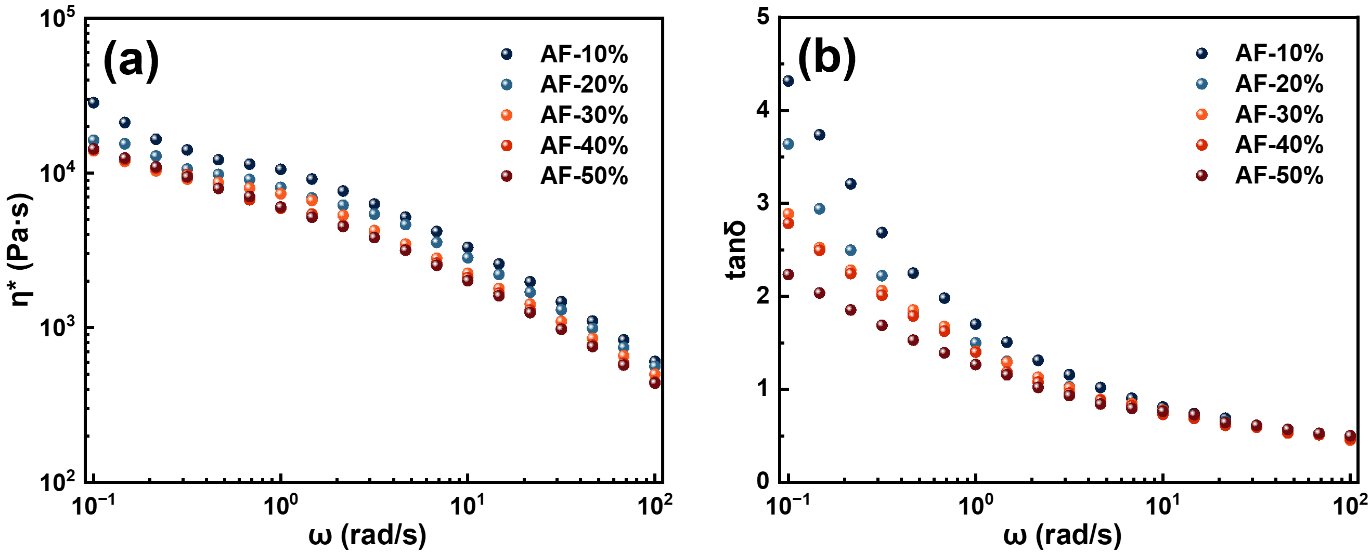
**

**Fig. S5 (a) Complex viscosity and (b) loss tangent as functions of frequency from 0.1-100 rad/s at 190 ^o^C for PMMA and PMMA/PVDF blends.**

Measuring the rheological behavior of blends with varying PVDF content can effectively analyze the viscoelastic changes in the PMMA/PVDF system, allowing for the prediction or assessment of the foaming behavior of material. **Figure S5(a)** shows the variation in complex viscosity (*η^*^*) with angular frequency for PMMA and PMMA/PVDF blends. Since PVDF has lower viscoelasticity compared to PMMA [1] at the same temperature, the complex viscosity of the blend decreases as the PVDF content increases. This phenomenon is primarily caused by two factors. Firstly, molten PVDF is more prone to orientation under shear, making chain slippage easier. Secondly, the addition of PVDF reduces the entanglement between PMMA molecular chains, weakening the intermolecular interactions and significantly plasticizing the system [1]. **Figure S5(b)** presents the loss angle tangent (tan *δ*) variation with angular frequency for PMMA and PMMA/PVDF blends. As the PVDF content increases, the tan *δ* of the blend decreases in the low-frequency region, indicating that the addition of PVDF transitions the blend system from a viscous to an elastic state. Additionally, as the molecular chains transition from an entangled state to a more disordered state, the sensitivity of the blend's viscoelastic response to shear rate decreases. This further confirms the strong plasticizing effect of PVDF on PMMA, which is beneficial for the solid-state temperature rising foaming of the polymer matrix.

**2.4 Blend Morphology**

To investigate the phase morphologies and dispersion states of PVDF and PMMA in the blends, the samples were analyzed via SEM (Nove NanoSEM450, Thermo Fisher Scientific Inc. Co., USA) and energy dispersive X-ray spectroscopy (EDS).


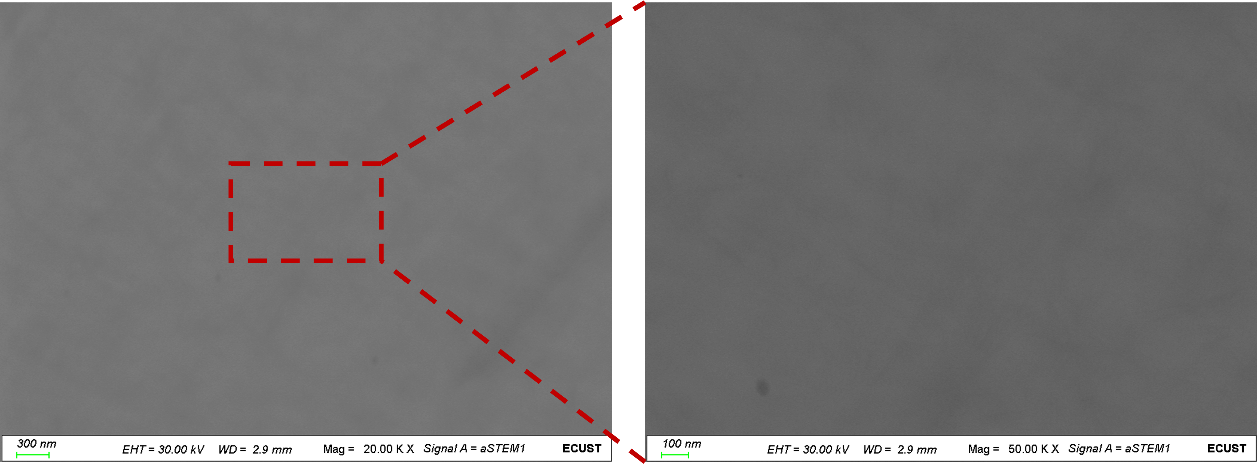


**Fig. S6 Crystalline Morphology (STEM images) of AF-40%.**

**2.5 Temperature Distribution of Microwave Heating**

An infrared thermal imager (H10, Hikvision, China) was employed to record the temperature distribution of PMMA and AF-50% under microwave radiation.

**3 Characterize of** **Foams**

**3.1 Structure of Foams**

The density (*ρ_f_*) of the foam was determined using an electronic densimeter (PMMD-A, Beijing Guance Jingdian Instrument Equipment Co., Ltd, China). The expansion ratio (*R_v_*) was calculated by means of **Equation (S6)**:

| $R_{v}=\frac{\rho_{i}}{\rho_{f}}$ | (S6) |
| --- | --- |

Where *ρ_i_* is the density of the unfoamed polymer.

SEM system was employed to visualize the morphology of foam. The sample is rapidly cooled using liquid nitrogen, followed by conducting a brittle fracture after a 10-minute immersion period. Subsequently, the fracture surface is examined using SEM. The Image-Pro Plus software (Version number: 6.0) is utilized to analyze SEM micrographs of the samples, allowing for the determination of the number average diameter (*D*) of all cells^2^, as calculated by **Equation (S7)**:

| $D=\frac{\sum d_{i}n_{i}}{\sum n_{i}}$ | (S7) |
| --- | --- |

Where *n_i_* is the number of cells with diameter *d_i_*. Cell density (*N*) and nucleation density (*N_0_*) are defined as the number of cells and nucleations of foaming polymer per cubic centimeter, which is calculated according to **Equation (S8-S9)** below:

| $N=\left( \frac{n}{A} \right)^{\frac{3}{2}}$ | (S8) |
| --- | --- |
| $N_{0}=N\times R_{v}$ | (S9) |

Where *n* is the total number of cells in the SEM micrograph, and *A* is the area of the SEM micrograph (unit is square centimeter, cm^2^).

Micro-computed tomography (micro-CT, Skyscan 1272, Bruker, Germany) with penetrative X-rays of 50 KeV and 200 μA was employed to visualize the morphology of foam and porosity distribution.

**3.2 Hydrophobicity and Water Absorption**

The contact angle between the water drop and the surface of the foams was tested by using a contact angle tester (JC 2000D, Powereach, China). To ensure the flatness of the sample surface, a sharp blade was used to cut the foams. The testing temperature was 23 °C and the volume of the water drop was 2 μL. The average value of the contact angle was taken from at least three places on the sample surface.

The foam was dried with dry air for 24 hours at room temperature. Subsequently, the foam was soaked in distilled water for 24 hours at room temperature. The surface water was wiped off with filter paper, and then weighed. The water absorption is calculated using the formula Q_t_ = (M_e_/M_r_)/M_0_, where Q_t_ is the molar amount of water absorbed by 100 g of the polymer composite, M_e_ is the mass of water at a given time, M_r_ is the relative molecular mass of water, and M_0_ is the initial mass of the sample.

**3.3 Chemical Resistance Testing**

The foam samples were kept in concentrated NaOH solution at PH=14, concentrated HCl solution at PH=0 and 1 mol/L NaCl solution for seven days, and changes in the size and morphology of the foam were observed before and after placement.

**3.4 Durability testing of foam**

The resistance of the foam to UV light and low temperatures was evaluated for a period of 7 days to assess any potential impact. The foam did not undergo any changes under these different conditions, indicating that it is practical.


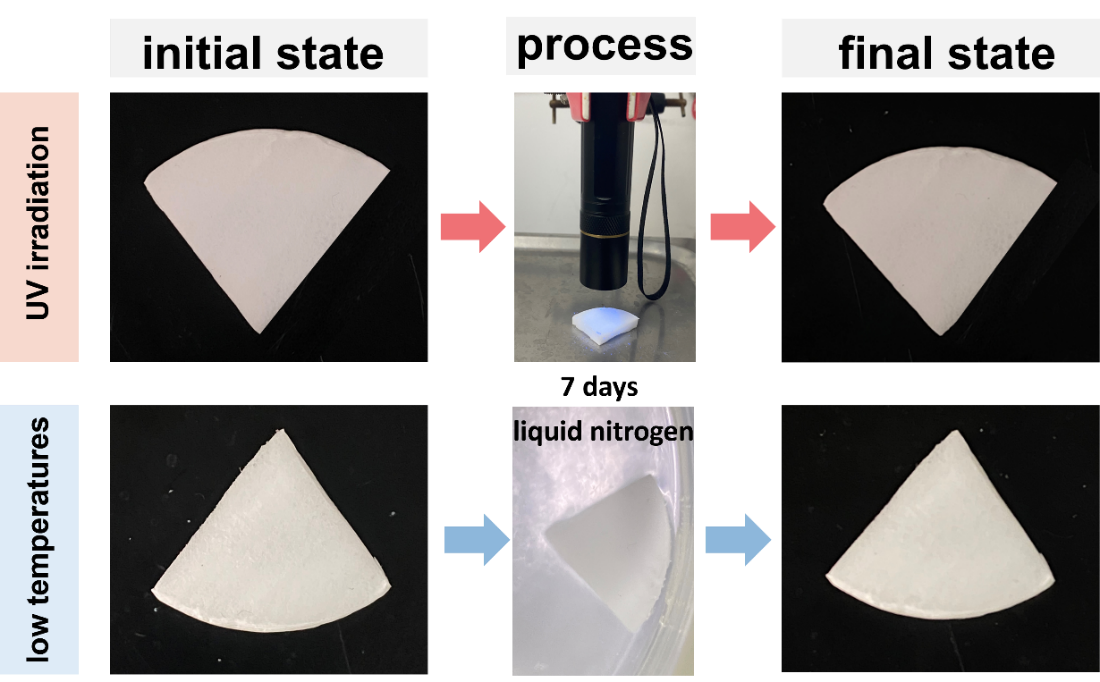


**Fig. S7 Conformational changes of PMMA/PVDF foams after 7 days of UV and low temperature treatment.**

**
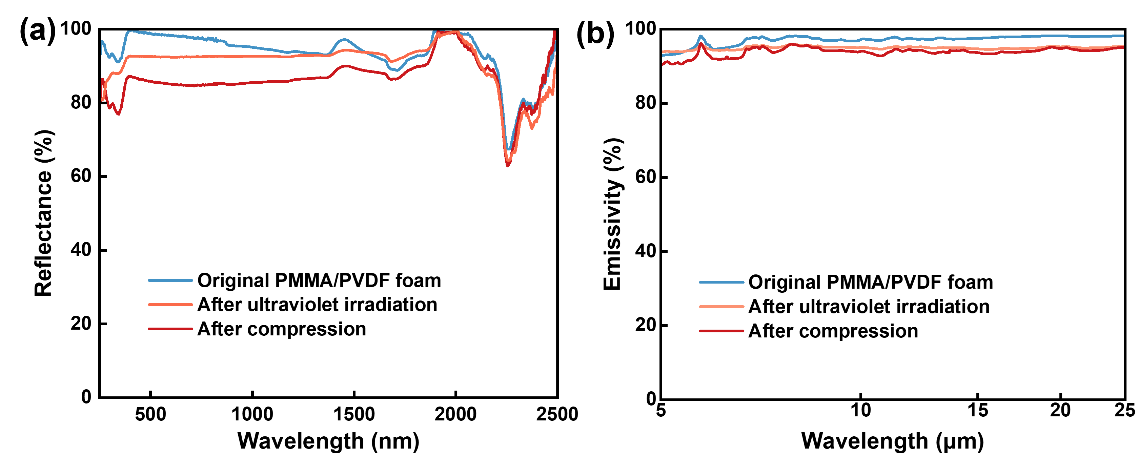
**

**Fig. S8 (a) Solar reflectance and (b) infrared emissivity of PMMA/PVDF foams subjected to UV radiation and compression**

**3.5 Compression Mechanics Testing**

Cyclic compression measurements of PMMA/PVDF foams were conducted utilizing a universal material testing machine (CMT-1104, Zhuhai SUST Electrical Equipment Co., Ltd, China). During the loading process, compression was applied at a controlled rate of 1 mm/min, reaching a maximum compressive strain of 50%. Subsequently, the samples were promptly released to their original equilibrium position with the same rate. This loading-unloading compression cycle was repeated 10 times for each sample to gauge the stability of their compression properties.

The compression elastic modulus (**Equation (S10)**) was calculated according to:

| $CM=\frac{F_{C}/A_{C}}{x_{C}/t_{C}}$ | (S10) |
| --- | --- |

Where *CM* is the compressive elastic modulus (MPa), *F_c_* is the compression force within the proportional limit (N), *A_c_* is the area of foams (mm^2^), *x_c_* is the deformation corresponding to *F_c_* (mm), *t_c_* is the thickness of foams (mm).

Compression strength is the compression stress when the foam is compressed to 50 %.

Energy loss coefficients (*ΔU/U*, **Equation (S11)**) was calculated from the obtained stress-strain curves.

| $\Delta U/U=\frac{A_{\text{loading }}-A_{\text{ulloading }}}{A_{\text{loading }}}\times100\%$ | (S11) |
| --- | --- |

Where *A_loading_* and *A_unloading_* represent the regions enclosed by the stress–strain curve during the loading and unloading processes, respectively. These areas are indicative of the energy absorption and energy release that occur during cyclic compression processes.

In the Poisson's ratio test, foam samples were cut into cubes measuring 10 × 10 × 4 mm. A line was drawn on the foam sample to mark the test point, which was perpendicular to the direction of tension or compression. The original length (*l_0_*) and thickness (*h_0_*) of the sample were measured using a vernier caliper. After compression, the length (*l*) and thickness (*h*) of the foam at different strains were recorded. The strain in the length direction (*ε_z_*) was calculated using the formula *ε_z_* = (*l* - *l_0_*) / *l_0_*, and the strain in the thickness direction (*ε_x_*) was calculated as *ε_x_* = (*h* - *h_0_*) / *h_0_*. The Poisson's ratio (*v*) of the foam sample was then determined using **Equation (S12)**. The Poisson's ratio of the PMMA/PVDF foam was taken as the average of three measurements.

| $v=-\frac{\varepsilon_{x}}{\varepsilon_{z}}$ | (S12) |
| --- | --- |

**3.6 Thermal Insulation Property**

Thermal conductivity was measured at room temperature by a transient plane source hot disk thermal constants analyzer (TPS 2500 S, Thermal Test Inc., Sweden). Each sample's thermal conductivity was measured 5 times with a deviation of less than 5%. In order to conduct the tests, a circular flat sensor with a radius of 2.001 mm was placed between two foam samples as a heat source and a temperature sensor. The power output, test duration, and detection depth were set to 5 mW, 5 s, and 5 mm. The foam was left at room temperature for more than three weeks before measurements to ensure that CO_2_ in the sample had diffused into the atmosphere. Measurements were carried out at room temperature using two square samples of approximately 5 mm thickness. The thermal conductivity of the gas, the thermal conductivity of the solid, and the percentage of thermal radiation were calculated for each sample using **Equation (S13)**:

| $K_{t}=K_{s}+K_{g}+K_{r}$ | (S13) |
| --- | --- |

where the thermal conductivity of the foam material Kt consists of three components, *K_s_* is the solid thermal conductivity, *K_g_* is the gaseous thermal conductivity, and *K_r_* is the radiative thermal conductivity.

The thermal conductivity of solids Ks could be calculated by **Equation (S14)**:

| $K_{s}=(1-\varphi)\left( \frac{2-f_{st}}{3} \right)K_{s,0}$ | (S14) |
| --- | --- |

where *K_s,0_* is the thermal conductivity of the blends and it can be calculated by **Equation (S15)**:

| $K_{s,0}=wK_{s,PMMA}+(1-w)K_{s,PVDF}-0.72w(1-w)\left( K_{s,PVDF}-K_{s,PMMA} \right)$ | (S15) |
| --- | --- |

where *w* is the PMMA content, *K_s,PMMA_* is the thermal conductivity of PMMA, 203.5 mW/(m·K), and *K_s,PVDF_* is the thermal conductivity of PVDF, 207.6 mW/(m·K)^3^.

The gas thermal conductivity *K_g_* can be calculated by **Equation (S16)**:

| $K_{g}=\varphi K_{g,eff}$ | (S16) |
| --- | --- |

where *K_g,eff_* is the thermal conductivity of a bulk gas and it can be calculated by **Equation (S17)**:

| $K_{g,eff}=\frac{K_{g,0}}{1+2K_{n}\beta}$ | (S17) |
| --- | --- |

where *K_g,0_* is the intrinsic thermal conductivity of gas, and *K_n_* is the Knudsen number. *β* is a parameter that describes the energy transfer caused by collisions between the gas molecules and the cell walls, usually taking value of 2. *K_n_* can be calculated by **Equation (S18)**:

| $K_{n}=\frac{\Lambda_{g}}{d_{c}}$ | (S18) |
| --- | --- |

where *Λ_g_* is the mean free path of gas molecules, usually taking value of 69 nm, *d_c_* is the cell size.

The radiative thermal conductivity can be calculated by **Equation (S19)**:

| $K_{r}=\frac{16n^{2}\sigma T^{3}}{3K_{e,R}}$ | (S19) |
| --- | --- |

where *n* is the effective index of refraction, 1, *σ* is Stefan-Boltzmann’s constant, 5.67×10^-8^ W/(m^2^∙K^4^), *T* is the temperature, *K_e,R_* is the Rosseland extinction coefficient and it can be estimated by **Equation (S20)**:

| $K_{e,R}=5.6712\times{10}^{6}d_{c}^{0.4264}(1-\varphi)$ | (S20) |
| --- | --- |

The temperature distribution of the foam on the heating table was recorded using an infrared thermal imager.

**3.7 Passive Daytime Radiant Cooling Performance**

The UV-Vis-NIR reflectance spectra (0.3-2.5 µm) was recorded using a spectrophotometer (Shimadzu UV-3600, Shimadzu Corporation, Japan) with an integrating sphere. The mid-infrared emittance spectra was measured by a Fourier Transform Infrared (FTIR) spectrometer (Nicolet IS50, Thermo Fisher Scientific Inc. Co., USA) with a diffuse integrating sphere.

Based on the principle of energy conservation, the net daytime radiative cooling power *P_R_* of the sample is expressed as:

| $P_{\text{R}\text{ }}(T)=P_{\text{rad }}(T)-P_{\text{atm }}(T)-P_{\text{sun }}-P_{\text{cond }+\text{ conv }}$ | (S21) |
| --- | --- |

where the radiative power of the sample is

| $P_{\text{rad }}(T)=A\int d\Omega cos\theta\int_{0}^{\infty} d\lambda I_{BB}(T,\lambda)\varepsilon(\lambda,\theta)$ | (S22) |
| --- | --- |

where $\int d\Omega=2\pi\int_{0}^{\pi/2} d\theta sin\theta$ is the angular integral over a hemisphere; $I_{\mathrm{BB}}(T,\lambda)=\frac{2hc^{2}}{\lambda^{5}}\frac{1}{e^{hc/\left( \lambda k_{s}T \right)}-1}$ is the radiance of a blackbody at temperature *T* (*T* = 303 K), *k_S_* is the Boltamann constant, *c* is the speed of light, and *λ* is the wavelength; *ε*_aw_(*λ*) is the emissivity of the sample in the atmospheric window; *A* is the surface area of radiative cooler. The absorbed power due to solar irradiation is

| $P_{\text{atm }}\left( T_{amb} \right)=A\int d\Omega cos\theta\int_{0}^{\infty} d\lambda I_{BB}\left( T_{amb,}\lambda\right)\varepsilon(\lambda,\theta)\varepsilon_{atm}(\lambda,\theta)$ | (S23) |
| --- | --- |

where *P_atm_* (*T_amb_*) represents absorbed power due to incident atmospheric thermal radiation. $\varepsilon_{atm}(\lambda,\theta)=1-\tau(\lambda)^{1/\cos\theta}$, where *τ*(*λ*) is the atmospheric transmittance in the zenith direction (air mass of 1.5 and water vapour column of 5.0 mm).

| $P_{\text{sun}\text{ }}=A\int_{0}^{\infty} d\lambda\varepsilon\left( \lambda,\theta_{\text{s}\text{un}} \right)I_{AM1.5}(\lambda)$ | (S24) |
| --- | --- |

where *P_sun_* is the absorbed power by the film from incident solar irradiance and *I_AM1.5_*(*λ*) is the standard solar irradiance.

| $P_{\text{cond }+\text{ conv }}\left( T,T_{amb} \right)=Ah_{c}\left( T_{amb}-T \right)$ | (S25) |
| --- | --- |

where *h_c_* is combined conduction and convection heating, which is set as 0, 3, 6, 9 and 12 W/m^2^·K in this study.

The actual heat flow density *q* (w/m^2^) per unit area of the radiant cooler is:

| $q=\varepsilon\delta\left( {T_{amb}}^{4}-T^{4} \right)$ | (S26) |
| --- | --- |

where *δ* is Stefan-Boltzmann constant.


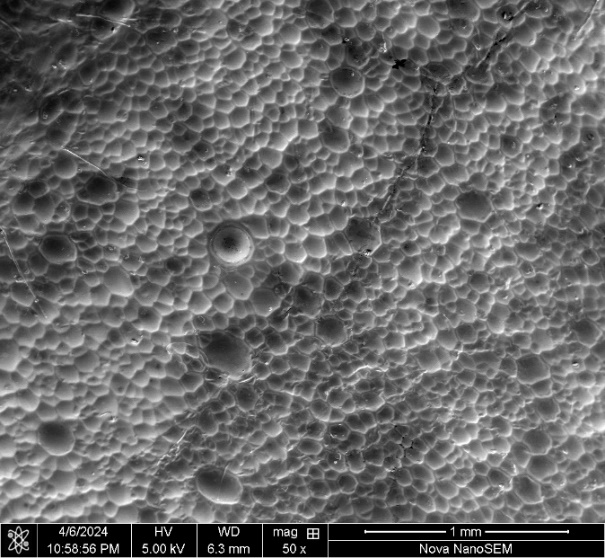


**Fig. S9 Morphology of Foam Skin Layers.**

**3.8 Recyclability**

The foam was cut into small pieces and then hot pressed through a hot press at 200 °C for 10 min at a pressure of 10 MPa, and the resulting samples were repeatedly foamed according to the previous conduction-microwave hybrid heating-assisted foaming process.


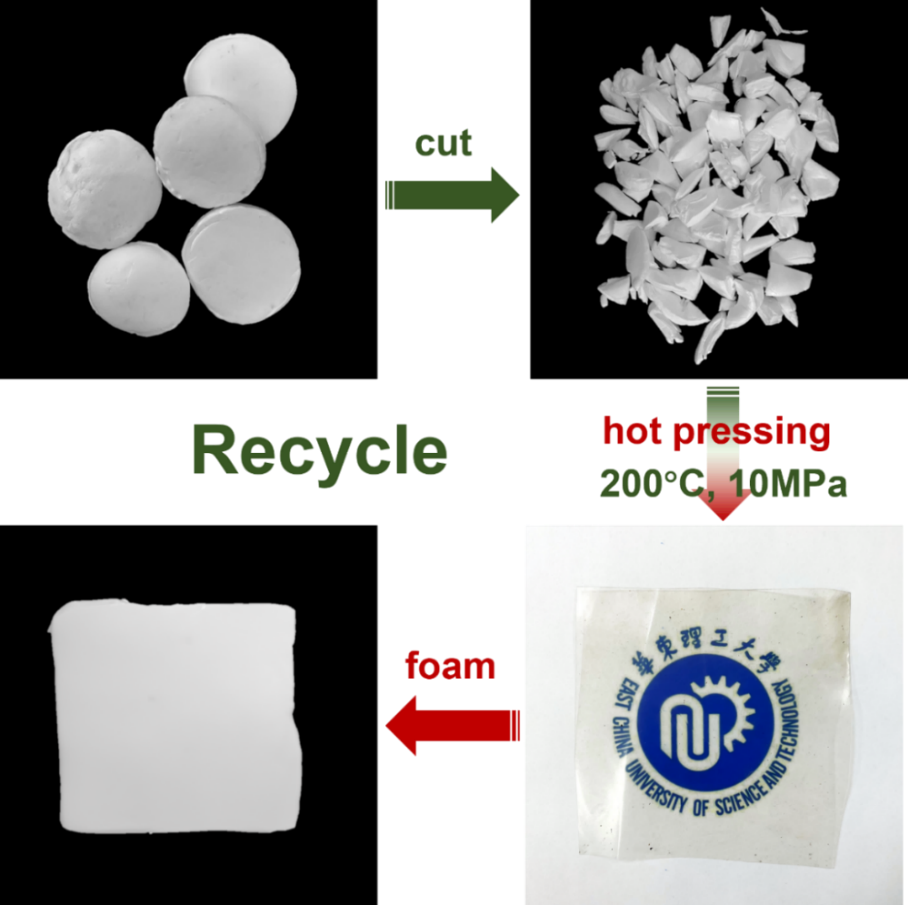


**Fig. S10 Recycling Process for PMMA/PVDF Foam.**

**4 Process Advantages and Challenges**

In traditional temperature-raising foaming, polymers require a longer time to reach the appropriate temperature, during which a significant amount of foaming agent may be consumed, leading to suboptimal foaming results. Additionally, the heat transfer from the outside to the inside can cause uneven heating, making it difficult to produce thicker foamed materials. In contrast, with conductive-microwave synergistic heating, the heating rate is faster, and the temperature distribution is more uniform. This avoids the issue of the center temperature being lower than the edge temperature, which is common in traditional heating methods, ensuring a more uniform and complete foaming process. As a result, foams produced using microwave-assisted foaming technology exhibit higher expansion ratios and better foaming performance.

Similar to other materials, the primary challenge in the microwave-assisted production of PMMA/PVDF foams lies in equipment considerations. In industrial applications, microwave heating and foaming equipment should focus on ensuring internal uniformity, which primarily depends on improvements to the equipment.

Simulation and modeling technologies are the best approaches to address these challenges. Developing more precise and reliable simulation and modeling tools can significantly assist in designing and optimizing microwave heating systems, as well as predicting heating effects. By considering the multiphysical interactions involved in the microwave heating process, such as electromagnetic field propagation, temperature distribution, and material phase changes, in-depth theoretical research and new method explorations can be conducted. These simulation and modeling tools allow engineers and researchers to simulate the behavior of microwave heating systems under various conditions, leading to a deeper understanding of the complex interactions and dynamic processes within the system. By integrating electromagnetic field solvers, heat transfer models, and material property databases, accurate predictions of temperature distribution, heating rates, and overall system performance can be achieved. As a result, more rational microwave equipment can be independently designed or integrated with devices utilizing other heating methods.

However, the industrial scaling of microwave processes remains a major obstacle to their widespread application. Therefore, future work should focus on improving microwave equipment to overcome these challenges and promote its application in industrial production.

**Reference**

1. Shi, Z.; Zhao, G.; Zhang, L.; Wang, G., Lightweight, strong, flame-retardant PVDF/PMMA microcellular foams for thermal insulation fabricated by supercritical CO2 foaming. *Composites Part B: Engineering* **2022,** *230*, 109554.

2. Zhong, W.; Hu, D.; Jia, X.; Huang, Y.; Wang, Y.; Lei, K.; Jiang, X.; Yu, J.; Chen, Y.; Zhao, L., A novel semi-continuous preparation mode of ultra-low density thermoplastic polyurethane foam. *Chemical Engineering Journal* **2024,** *481*, 148402.

3. Shi, Z.; Zhao, G.; Wang, G.; Zhang, L.; Wei, C.; Chai, J., Development of ultralight, tough and hydrophobic polymethylmethacrylate/polyvinylidene fluoride shape memory foams for heat insulation applications. *Materials & Design* **2023,** *225*, 111527.
